# Volatiles of symbiotic bacterial origin explain ectoparasitism and fledging success of hoopoes

**DOI:** 10.1186/s42523-024-00312-9

**Published:** 2024-05-10

**Authors:** Mónica Mazorra-Alonso, Juan Manuel Peralta-Sánchez, Manuel Martín-Vivaldi, Manuel Martínez-Bueno, Rafael Núñez Gómez, Juan José Soler

**Affiliations:** 1 Departamento de Ecología Funcional y Evolutiva, Estación Experimental de Zonas Áridas (CSIC), Almería, Spain; 2 Departamento de Zoología, Universidad de Sevilla, Seville, Spain; 3 Departamento de Zoología, Universidad de Granada, Granada, Spain; 4 Departamento de Microbiología, Universidad de Granada, Granada, Spain; 5 Unidad Asociada (CSIC): Coevolución: Cucos, Hospedadores y Bacterias Simbiontes. Universidad de Granada, Granada, Spain; 6 Servicio de Instrumentación Científica, Estación Experimental del Zaidín (CSIC), Granada, Spain

**Keywords:** Animal odour, Avian microbiota, *Carnus hemapterus*, Eavesdropping parasites, Fermentation hypothesis, Uropygial secretion

## Abstract

**Background:**

Some parasites use olfactory cues to detect their hosts and, since bacterial symbionts are partially responsible for animal odours, they could influence host parasitism. By autoclaving nest materials of hoopoe (*Upupa epops*) nests before reproduction started, we explored the hypothetical links between host-associated bacteria, volatiles and parasitism. During the nestling stage, we (i) estimated the level of ectoparasitism by chewing lice (Suborder Mallophaga) in adult hoopoe females and by *Carnus haemapterus* flies in nestlings, and (ii) characterized microbial communities and volatile profiles of nest environments (nest material and nest cavity, respectively) and uropygial secretions.

**Results:**

Experimental nests had less diverse bacterial communities and more diverse volatile profiles than control nests, while occupants experienced lower intensity of parasitism in experimental than in control nests. The experiment also affected beta diversity of the microbial communities of nest material and of the volatiles of the nestling uropygial secretions. Moreover, microbial communities of uropygial secretions and of nest materials covaried with their volatile profiles, while the volatile profile of the bird secretions explained nest volatile profile. Finally, a subset of the volatiles and bacteria detected in the nest material and uropygial secretions were associated with the ectoparasitism intensity of both adult females and nestlings, and with fledging success.

**Conclusions:**

These results show that a component of animal odours is linked with the microbial communities of the host and its reproductive environment, and emphasize that the associations between bacteria, ectoparasitism and reproductive success are partially mediated by volatiles of bacterial origin. Future work should focus on mechanisms underlying the detected patterns.

**Supplementary Information:**

The online version contains supplementary material available at 10.1186/s42523-024-00312-9.

## Background

Symbiotic microorganisms are essential for understanding the evolution and functioning of their animal hosts [1–4]. For instance, bacterial symbionts are partly responsible for the emission of volatiles that contribute to animal odours [1, 5], and then these microorganisms may play key roles in animal communication [6–9].

The possible role of bacteria contributing to animal odours and informing on animal characteristics was first posited within the fermentation hypothesis [10, 11]. This hypothesis was initially restricted to odours derived from mammalian secretions used in chemical communication, but it is currently extended to secretions of other animal taxa [6, 7]. Exocrine glands that open out of the body greatly determine their volatile profile (i.e. odour) of animals [12–17] and, since glands provide suitable environments for bacterial growth [8, 18–21], odour profile could be partially produced by bacteria that inhabit those glands. The only exocrine gland birds have in the skin is the uropygium [22, 23], which produces secretion rich in volatiles [24] that, at least partially, could be by-products of the metabolism of bacterial symbionts [25]. In accordance, hoopoes (*Upupa epops*) [25] and dark-eyed juncos (*Junco hyemalis*) [26] host bacteria in their uropygial glands that produce key volatiles that are apparently involved in chemical social communication [8, 26, 28, but see 29]. Therefore, a relationship between diversity and composition of bacterial communities and volatile profiles of the uropygial secretion is therefore a key prediction of the hypothesized role of bacteria in animal odours. Evidences supporting this prediction has been found in different taxa, including mammals [19, 21], insects [5, 30–32] and amphibians [33]. In birds, the role of microorganisms mediating the odours of avian nests or uropygial secretions is a rapidly growing field of research [28, 29, 34, 35].

The hypothetical informative value of volatiles of bacterial origin implies that they might be involved in animal communication, either as signals [sensu 36], or as Inadvertent Social Information [ISI, sensu 37] that would be of interest for con- and hetero-specifics [7]. Communities of symbiotic bacteria, including the gut microbiota, are usually related to phenotypic condition, immune-state, physiology and behaviour of their animal hosts [3]. Then, particularities of the volatile profiles of bacterial origin would inform on these or other host traits. Accordingly, particular volatiles of bacterial origin are related to host sex, age, social status, or even group membership of individuals in some taxa [19, 27, 28, 38], which is valuable information for interacting conspecifics. In addition, hetero-specifics ectoparasites and predators frequently use animal odours as cues to detect or select their hosts [39–43]; some of those odours are possibly produced by bacterial symbionts [1, 2, 7, 9]. In accordance with this scenario, the experimental modification of the microbiota of nest materials affects the volatile profiles of great tit (*Parus major*) nests [44], the intensity of *Carnus hemapterus* ecto-parasitism suffered by hoopoe nestlings [45], and the probability of nest predation experienced by spotless starling (*Sturnus unicolor*) nestlings [46]. Volatiles of symbiotic bacterial origin might also act as repellent of ectoparasites and predators, indicating that their effects on host-parasite interactions are complex [47]. For instance, depending on the considered skin bacteria of humans and their produced volatiles, they can attract or repel mosquitoes [48, 49]. This scenario leads to the hypothesis that volatiles produced by bacterial communities are partially determining the intensity of parasitism and/or the probability of predation of their animal hosts. Therefore, host traits favouring bacterial symbionts that reduce the strength of selection pressures imposed by predators and parasites on their hosts would be adaptive [50].

The hoopoe is one of the few species where a link between the complex symbiotic bacterial community present in their uropygial gland [51–53] and the volatile components of their secretion has been experimentally demonstrated [25]. Moreover, hoopoes do not build nests, but prefer to re-use nest cavities with soft-material remains from previous reproductive events of conspecifics or heterospecifics [54]. The mutualistic association between hoopoes and the antibiotic-producing bacteria of their uropygial gland only appears during the nesting phase (i.e. within nest cavities) [55], and nest material from previous reproduction affects the bacterial community of the uropygial gland [54]. Finally, autoclaving the old-nest material before reproduction affects both the density of bacteria in hoopoe nests and the intensity of ectoparasitism suffered by nestlings [45]. However, current knowledge lacks for evidences supporting the expected link between the effects of volatiles produced by microbial symbionts and risk of parasitism in nesting birds, including hoopoes.

The hypothetical role of chemicals of bacterial origin determining parasitism in hoopoes assumes that bacterial communities and volatiles profiles of particular environments should be related to each other. Then, we first explore whether particularities of the microbiota of the nest environment (i.e., nest material and uropygial secretion of females and nestlings) associated with volatile profiles of the nest environment and of the uropygial secretions of adult females and nestlings. Moreover, we also explore whether volatiles of the nest environment and those of the uropygial secretion of nesting birds (adult females and nestlings) associated to each other, which will shed light on the hypothetical contribution of animal secretion volatiles on the general odour of nest environment that parasites might use to detect host nests. Adult females rarely leave the nest during the incubation and hatching periods (8–10 days after the first egg hatches) [56], while the preen gland of nestlings usually starts to produce secretion on the sixth to seventh day after hatching [55]. Thus, we tested those assumptions with samples collected from hoopoe nests, females and nestlings at the beginning of the nestling period, and from hoopoe nests and nestlings at the end of the nestling stage.

Taking advantages of current knowledge in hoopoes, we hypothesized that volatiles produced by bacterial communities are, at least partially, determining the intensity of parasitism of their host. For this purpose, we experimentally manipulated the bacterial environment of hoopoe nests by installing new nest-boxes added with old nest material that were (experimental) or were not (control) previously autoclaved. We explored the experimental effects on bacterial communities and volatile profiles of nest material and uropygial secretion of adult females and nestlings. We estimated intensity of parasitism of nestling and adult females by the hematophagous fly *C. hemapterus* and by chewing lice, respectively. *C. hemapterus* is the most abundant ectoparasite of hoopoe nestlings in our study area [51, 66], while chewing lice (Suborder Mallophaga) are frequently detected in adult nesting females, but not in nestlings. As *C. hemapterus* likely use odours to search and locate active nests [57], and its parasitism likely affect fledging success [58], we explored the association between diversity of the bacterial communities and of the volatile profiles of the nest environments and ectoparasitism and fledging success of hoopoes.

## Results

### Bacteria and volatiles from nest material and uropygial secretions

The sequencing of the bacterial community of nest material produced 11,566,822 sequences and 2,481,899 were retained in the ASV table after filtering (number of samples = 144, average number of sequences per sample (min, max) = 17,235.41 (5000, 51,256)). The sequencing of uropygial secretion samples produced 17,866,248 sequences and 10,755,653 were retained in the ASV table after filtering (number of samples = 344, mean average number of sequences per sample (min, max) = 31,266.43 (8,723, 64,867)). The number of collected bacterial samples from experimental, control and natural nests is listed in the Additional file 1: Table S1.

153 ASVs were retained in nest material samples and 50 ASVs in uropygial secretion samples for subsequent analyses.

**Fig. 1:**
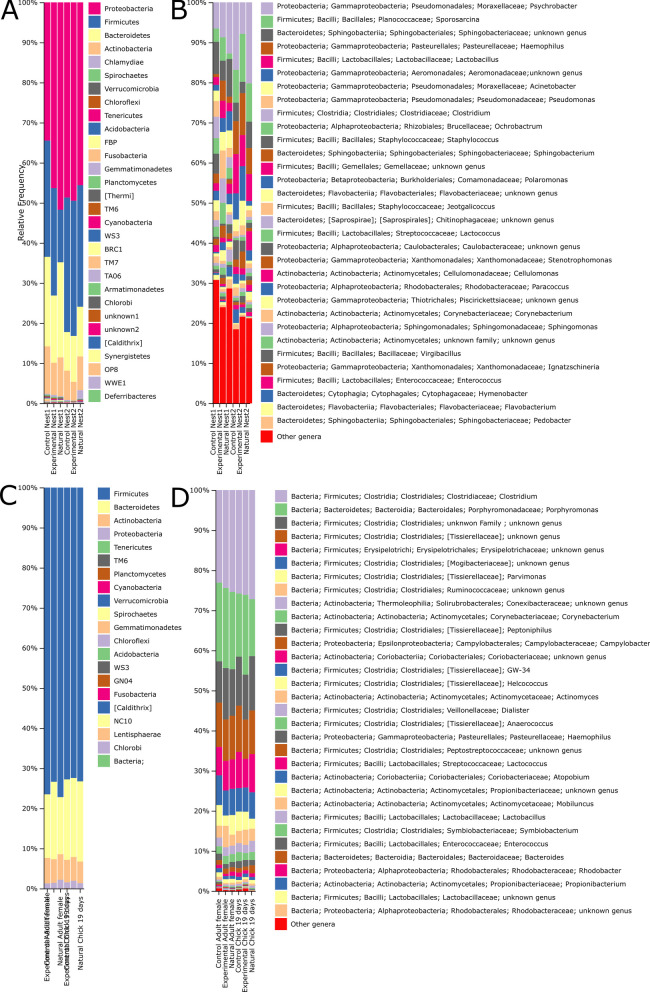
Microbial composition at the phylum and the genus levels of nest materials (**A** and **B**), and of adult female and nestling secretions (**C** and **D**) collapsed by experimental treatment at different nestling periods (1: 6–8 days old nestlings; 2: 17 days-old nestlings). While in experimental nest, nest material was autoclaved before the reproduction started and placed in new nest boxes, in control ones, the nest material was not autoclaved and placed in new nest boxes. In natural nests, old nest boxes and nest material were not manipulated. Taxa in the legend are sorted from the most to lowest abundant

At the phylum level, nest material was dominated by Proteobacteria, followed by Firmicutes, Bacteroidetes and Actinobacteria at both nestling stages (Fig. 1A). At the genus level, *Psychrobacter* resulted the most dominant bacteria genus in nest samples, although minority ASVs were abundant (between 18 and 31%, Fig. 1B).

Firmicutes were the phylum with the highest relative abundance in secretion samples, followed by Bacteroidetes and Actinobacteria (Fig. 1C). Accordingly, *Clostridium* and other genera belonging to Order Clostridiales presented the highest relative abundances in these samples (Fig. 1D).

**Fig. 2:**
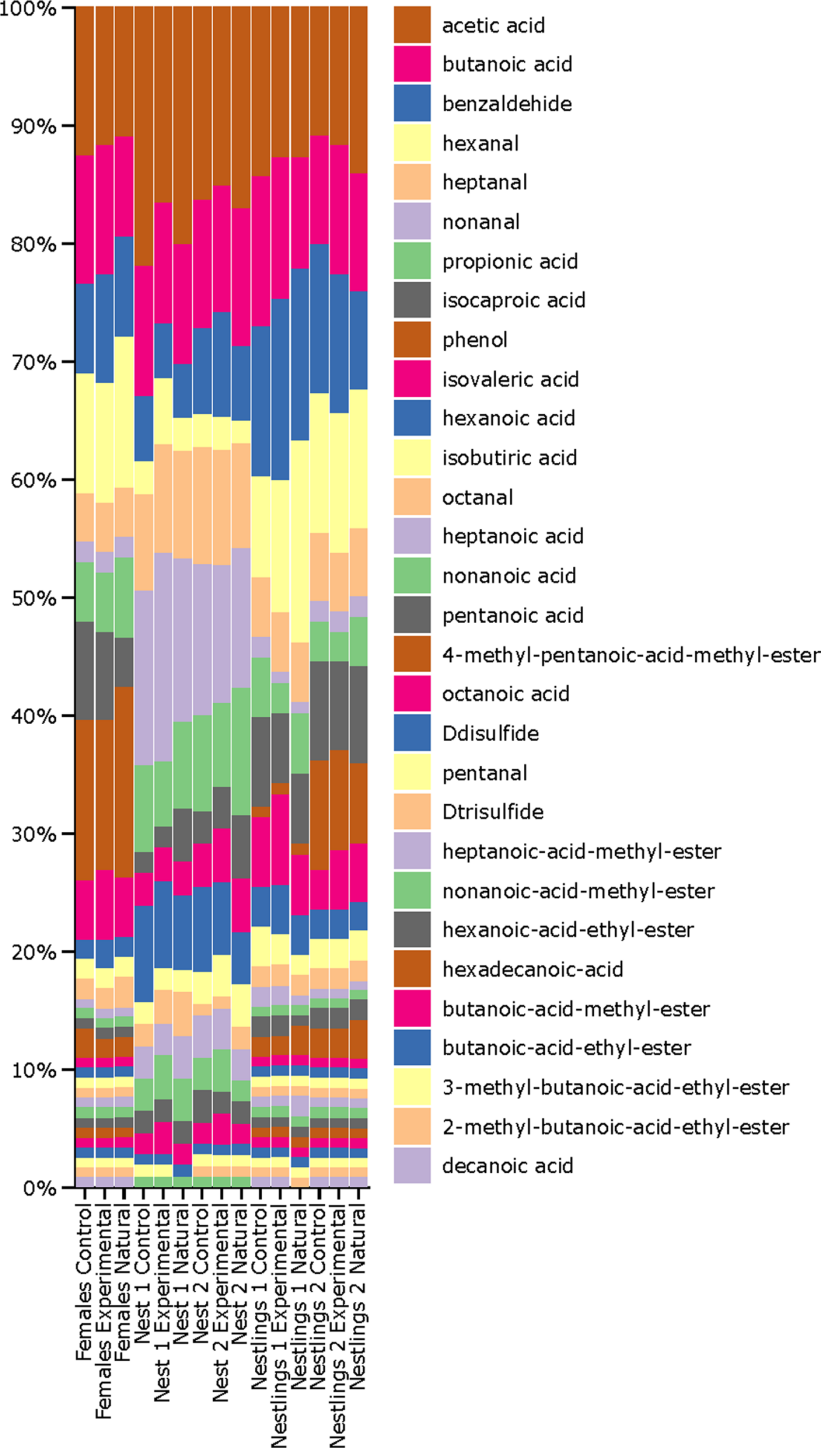
Volatile profile of nest materials and of adult female and nestling secretions collapse by experimental treatment at different nestling periods (1: 6–8 days old nestlings; 2: 17 days-old nestlings). While in experimental nest, nest material was autoclaved before the reproduction started and placed in new nest boxes, in control ones, the nest material was not autoclaved and placed in new nest boxes. In natural nests, old nest boxes and nest material were not manipulated

Acetic and butanoic acid and benzaldehyde were the most abundant volatiles in all samples (Fig. 2), and the relative abundance of the rest of volatiles depended of the type of sample. Some aldehydes as hexanal, heptanal and nonanal resulted relatively more abundant in nest samples, while isocaproic, propionic and isovaleric acids and phenol were relatively more abundant in secretion samples of females and nestlings (Fig. 2).

### Effects of autoclaving nest material in bacterial communities and volatile profiles of the nest environment

**Fig. 3:**
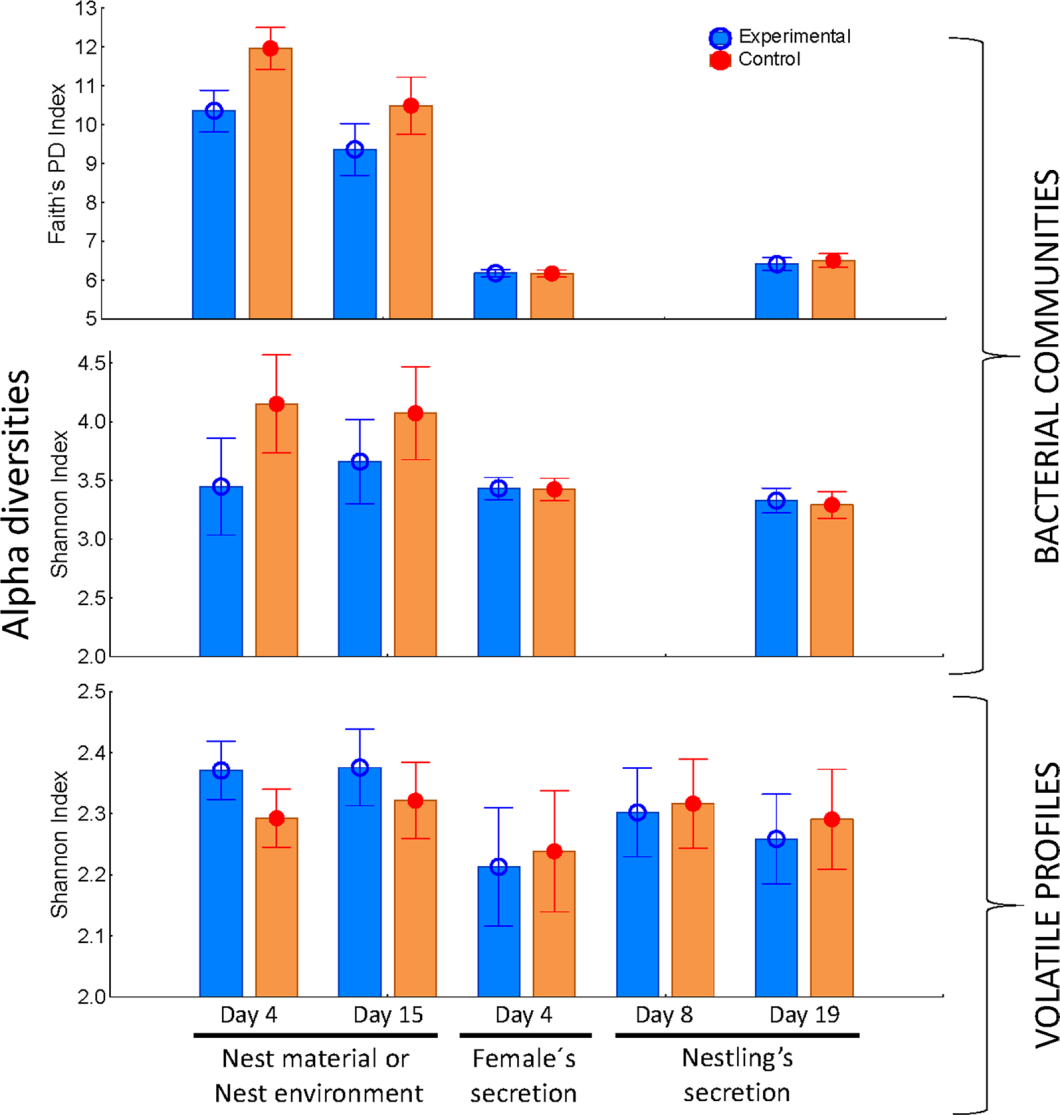
Alpha diversity of the bacterial communities (Shannon and Faith’s phylogenetic diversity (PD) indexes) and of volatile profiles (Shannon index) of samples collected in experimental (old nest material from previous reproduction was autoclaved and used to fill new nest boxes before reproduction started) and control nests (old nest material from previous reproduction was directly used to fill new nest boxes). Analysed samples included volatiles from nest environment (days four and 15 after hatching), and from female (Day 4 after hatching) and nestling (days 8 and 19 after hatching) uropygial secretions, and bacterial communities from the nest material (days four and 15 after hatching) and from the secretion of females and nestlings. Values are means ± 95% CI

**Fig. 4:**
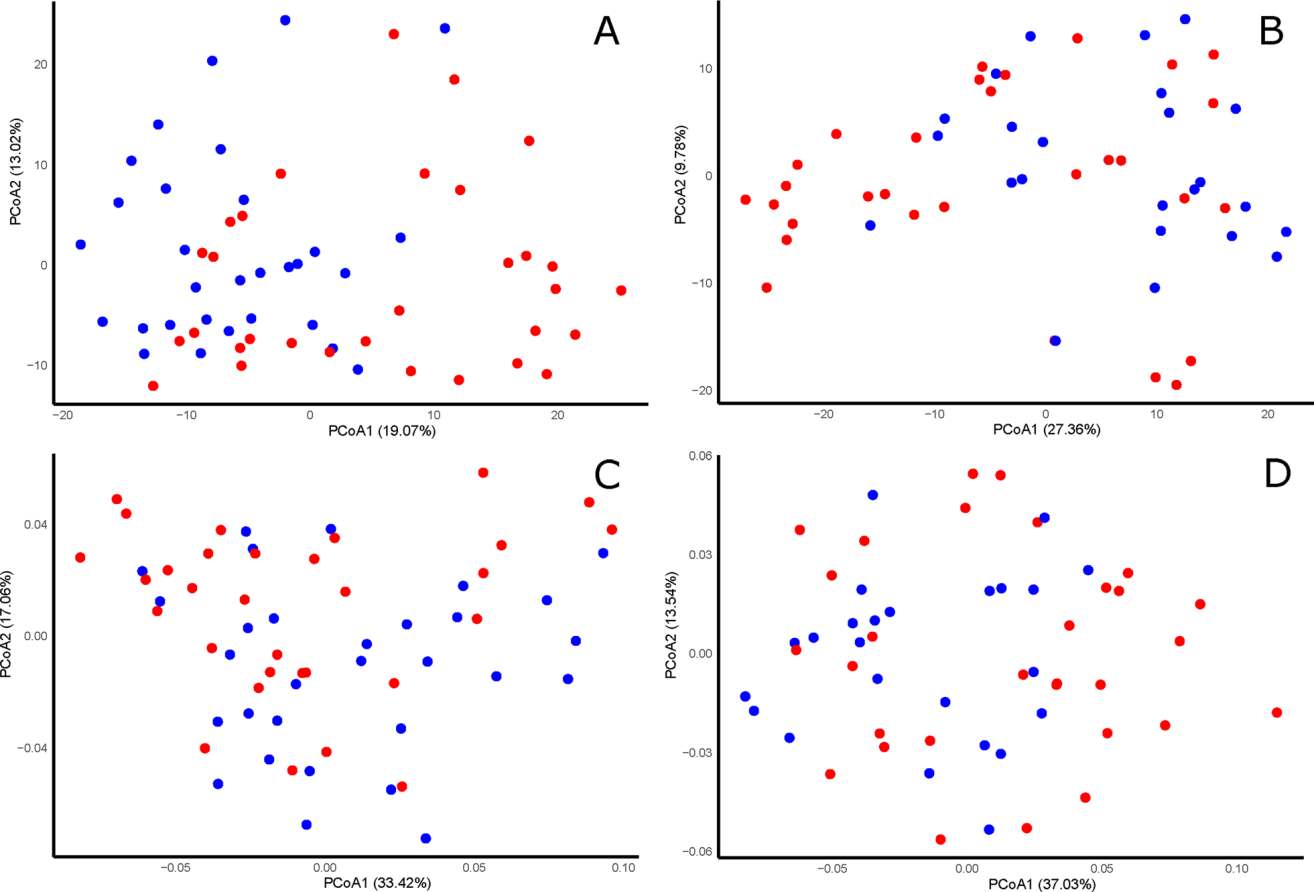
Principal coordinate analyses of the bacterial communities of experimental (red) and control (blue) nest materials of hoopoe nest-boxes. Samples collected at the beginning of the nestling stage were based in Aitchison (**A**) and PhilR distance (**B**). Similarly, samples collected at the end of the nestling stage were based in Aitchison (**C**) and PhilR distance (**D**). (**B** and **D**)

After controlling for the effect of laying date and study year, bacterial communities of nest material at the beginning (Shannon Index: F = 5.73, d.f. = 1,63, *P* = 0.020; Faith’s PD Index: F = 18.0, d.f. = 1,63, *P* < 0.001; Fig. 3) and at the end of the nestling stage (Shannon Index: F = 2.39, d.f. = 1,50, *P* = 0.129; Faith’s PD Index: F = 5.19, d.f. = 1,50, *P* = 0.027; Fig. 3) showed lower alpha diversity in experimental than in control nests (Additional file 1: Table S2). Both communities separated to each other (i.e., beta diversity) at the beginning, and at the end of the nestling period (for Aitchison and PhilR distance matrixes) (Table 1; Fig. 4).

**Table 1:** Results from PERMANOVAs exploring the effects of the experimental sterilization of nest materials before reproduction on beta-diversity indexes of bacterial communities (Aitchison and PhilR) and volatile profiles (Aitchison) of different sample types collected at different nestling ages (day)

	Experimental treatment			Study year			Laying date		
	*Pseudo F*	df	*p*	*Pseudo F*	df	*p*	*Pseudo F*	df	*p*
*Bacterial communities*									
*Nest material (day 4)*									
Aitchison	4.59	1,57	** < 0.001**	2.41	1,57	**0.002**	1.28	1,57	0.149
PhilR	3.47	1,57	**0.003**	6.02	1,57	** < 0.001**	1.23	1,57	0.240
*Nest material (day 15)*									
Aitchison	3.76	1,50	**0.001**	3.52	1,50	** < 0.001**	1.40	1,50	0.118
PhilR	4.49	1,50	**0.001**	7.54	1,50	** < 0.001**	1.55	1,50	0.122
*Female secretion (day 4)*									
Aitchison	1.24	1,74	0.173	1.00	1,74	0.458	1.18	1,74	0.230
PhilR	0.84	1,74	0.480	0.83	1,74	0.534	0.39	1,74	0.990
*Nestling secretion (day 19)*									
Aitchison	1.03	1,61	0.410	1.31	1,61	0.111	0.95	1,61	0.547
PhilR	0.57	1,61	0.753	0.67	1,61	0.643	2.44	1,61	**0.045**
*Volatile profiles*									
*Nest box (day 7)*									
Aitchison	1.54	1,55	0.129	23.68	1,55	** < 0.001**	5.34	1,55	** < 0.001**
*Nest box (day 18)*									
Aitchison	0.49	1,51	0.868	12.12	1,51	** < 0.001**	3.39	1,51	**0.004**
*Female secretion (day 4)*									
Aitchison	0.57	1,80	0.671	7.92	1,80	** < 0.001**	0.73	1,80	0.548
*Nestling secretion (day 8)*									
Aitchison	1.94	1,63	0.085	11.99	1,63	** < 0.001**	1.19	1,63	0.282
*Nestling secretion (day 19)*									
Aitchison	1.12	1,63	0.319	15.05	1,63	** < 0.001**	2.50	1,63	**0.041**

The models included study year (2017 *vs.* 2018) and laying date (early *vs.* late reproduction) as fixed factors. *P* values lower than 0.05 are in bold font

Nest materials of unmanipulated nest-boxes (i.e., natural nests) showed bacterial communities of lower alpha diversity than those of control nests (Additional file 1: Table S3) both at the beginning (Shannon Index: F = 2.92, d.f. = 1,42, P = 0.095; Faith’s PD Index: F = 36.0, d.f. = 1,42, *P* < 0.001) and at the end of the nestling stage (Shannon Index: F = 7.49, d.f. = 1,50, *P* = 0.010; Faith’s PD Index: F = 6.03, d.f. = 1,50, *P* = 0.020). Those bacterial communities also differed in terms of beta-diversity indexes (PERMANOVAs: early stage: Pseudo F > 3.85, d.f. = 1,42, *P* < 0.001; late stage: Pseudo F > 4.53; d.f. = 1,33, *P* < 0.001).

At the beginning of the nestling stage, *Staphylococcus spp.* was more abundant in nest materials of control nests than in those of experimental nests. *Staphylococcus spp.*, *Nocardia spp., Yaniella spp*. and an unknown genus belonging to the Order Lactobacillales were more abundant in control nests than in unmanipulated nests (ANCOM analyses, Table 2). At the end of the nestling period, the genera *Corynebacterium spp.* was more abundant in the material collected from control than experimental nests. *Yaniella spp.* was more abundant in materials of control nests than in those from unmanipulated nests (ANCOM analyses, Table 2).

**Table 2:** Pairwise differences in abundance of particular bacterial genus between experimental (E) and control (C) nests, and between each of them and unmanipulated (N-natural) nests

Nest material (day 4)			Nest material (day 15)		
E–C W ${\overline{\text{X}}}$s	E–N W ${\overline{\text{X}}}$s	C–N W ${\overline{\text{X}}}$s	E–C W ${\overline{\text{X}}}$s	E–N W ${\overline{\text{X}}}$s	C–N W ${\overline{\text{X}}}$s
*Staphylococcus* spp*.*					
**418** 992–6425	–	**442** 6425–200	–	–	–
*Nocardia* spp*.*					
–		**439** 2474–191	–	–	–
Unidentified genus of the order Lactobacillales					
–		**421** 989–141	–	–	–
*Yaniella* spp*.*					
–		**410** 936–69	–	–	**316** 344–88
*Corynebacterium* spp*.*					
–		–	**252** 851–652	–	–
Unidentified genus of the family Sphingonomadaceae					
–	**397** 96–403	**-**	–	–	–
*Sphingobium* spp*.*					
–	**384** 177–547	–	–	–	–
Unidentified genus of the order Sphingonomadales					
–	**383** 187–506	**–**	–	–	–
*Sphingomonas* spp*.*					
–	–	–	–	**283** 48–569	–
*Dyadobacters* spp.					
–	**339** 189–800	**–**	–	–	–

Statistical tests were separately performed for each bacterial community. For statistically significant comparisons, we show **W** values from ANCOM analyses, and mean abundance of bacterial genera in each compared group (${\overline{\text{X}}}$s). Only bacterial taxa for which we found statistically significant differences are shown

Volatile profiles of nest environment, at the early nestling stage, were significantly more diverse in experimental than in control nests (alpha diversity, GLM: *F* = 5.43, d.f. = 1,55, P = 0.023; Fig. 3 ALPHA), after controlling for the significant effects of study year and laying date (Additional file 1: Table S2). However, at that nestling stage, beta diversity of volatiles did not differ between experimental treatments (Table 1; Fig. 4). No differences in alpha or beta diversity was found at the end of the nestling stage (Additional file 1: Table S2; Fig. 3; beta diversity: Table 1, Fig. 4).

Similarly, when comparing volatile profiles of control and natural nests, statistical significant differences were not detected, independently of the sampling period (alpha diversity, early stage GLM: *F* = 1.51, d.f. = 1,28, *P* = 0.230; late-stage GLM: *F* = 0.27, d.f. = 1,26, *P* = 0.607; beta diversity, early stage PERMANOVAs: Pseudo *F* < 0.60, d.f. = 1,28, *P* > 0.750; late-stage PERMANOVAs for nestlings: Pseudo *F* < 0.60, d.f. = 1,26, *P* > 0.720).

Finally, the abundances of none of the detected volatiles differ significantly (*P* > 0.05) between treatments (ANCOM analyses, results no shown).

### Effects of autoclaving nest material in bacterial communities and volatile profiles of the uropygial gland secretions of nestlings and adult females

Autoclaving nest material before reproduction started did not induce changes on the microbiota of the uropygial secretion of nestlings or adult females. After taking into account the effects of study year and nestling stage, our experiment did not affect alpha (Shannon or Faith’s PD; *F*_1,74_ < 0.02, *p* > 0.896; Fig. 3; Additional file 1: Table S2) or beta diversity (Table 1), or the abundance of any of the detected bacterial genera (ANCOM analyses, Table 2) in the uropygial secretion of adult females. Similarly, the experiment neither affected alpha (Shannon or Faith’s PD, *F*_1,61_ < 0.53, *p* > 0.470; Additional file 1: Table S2) nor beta diversity of the bacterial community of the uropygial secretion of nestlings (Fig. 3; Table 1). Finally, the relative abundance of *Lactococcus spp.* was higher in experimental nests than control in control ones (ANCOM analyses, W = 199, experimental relative abundance = 3202, control ones = 1004).

Bacterial community of the uropygial gland secretion of adult females and nestlings from control and natural nests did not differ in terms of alpha (Shannon or Faith’s PD; adult females: F < 2.55, df = 1,53, *P* > 0.116; nestlings: F < 0.10, df = 1,39, *P* > 0.579; Additional file 1: Table S3) or beta diversity (PERMANOVAs for adult females: Pseudo *F* < 1.26, d.f. = 1,53, *p* > 0.167; PERMANOVAs for nestlings: Pseudo *F* < 1.30; d.f. = 1,39, *p* > 0.132). Only the relative abundance of *Propionibacterium spp.* was significantly higher in the female secretion of experimental nest than in female secretion of unmanipulated nests (ANCOM analyses, W = 30, experimental relative abundance = 290, unmanipulated ones = 8).

With respect to volatile profiles, estimated alpha and beta diversity of volatile profiles of the uropygial secretions of adult females or nestlings did not differ between experimental and control nests (Table 1; Additional file 1: Table S2).

Similarly, volatile profiles of the uropygial secretion of adult females or nestlings from control and natural nests did not significantly differ in terms of alpha (Additional file 1: Table S3) or beta diversity (PERMANOVAs: Pseudo *F* < 1.97, d.f. = 1,50, *p* > 0.087). However, abundance of some of the detected volatiles in adult female secretions differed significantly between experimental, control and natural nests (ANCOM analyses, Table 3).

**Table 3:** Pairwise differences in relative abundance of chemical volatiles between females in experimental (E) and control (C) nests, and between each of them and natural (N) nests, in female secretions

	Female secretion (day 4)		
	E–C W ${\overline{\text{X}}}$s	E–N W ${\overline{\text{X}}}$s	C–N W ${\overline{\text{X}}}$s
Esters			
Nonanoic acid methyl ester	–	**15** 1.07–1.64	**5** 1.09–1.64
Heptanoic acid mehyl ester	–	**13** 1.51–1.36	**2** 1.10–1.36
Butanoic acid ethil ester	–		**1** 1.26–1.00
Acids			
Nonanoic acid	–		**5** 1.04–1.18
Butanoic acid	–		**4** 12.38–9.16
Pentanoic acid	–		**4** 1.76–1.42
Isobutyric acid	–		**2** 2.58–1.90
Octanoic acid	–		**2** 1.04–1.29
Acetic acid	–		**1** 13.88–12.40
Isocaproic acid	–		**1** 10.99–4.96

For statistically significant comparisons, we show **W** values from ANCOM analyses, and mean relative abundance of volatiles in each compared group (${\overline{\text{X}}}$s). Only volatiles for which between-groups comparisons reached statistical significance are shown

### Associations between composition of the bacterial communities and the volatile profiles

Bacterial communities of the uropygial gland secretion of adult females (Mantel tests, R^2^ < 0.01, *p* = 0.771) or nestlings (Mantel tests, R^2^ < 0.01, *p* = 0.606) did not predict their respective volatile profiles. In spite of bacterial profiles of nest material at the beginning of the nestling period did not predict volatile profiles of nest environment (Mantel tests, R^2^ < 0.001, *p* = 0.317), bacterial profiles of nest material at the end of the nestling stage was correlated with its volatile profile (Mantel tests, R^2^ = 0.012, *p* = 0.037).

**Fig. 5:**
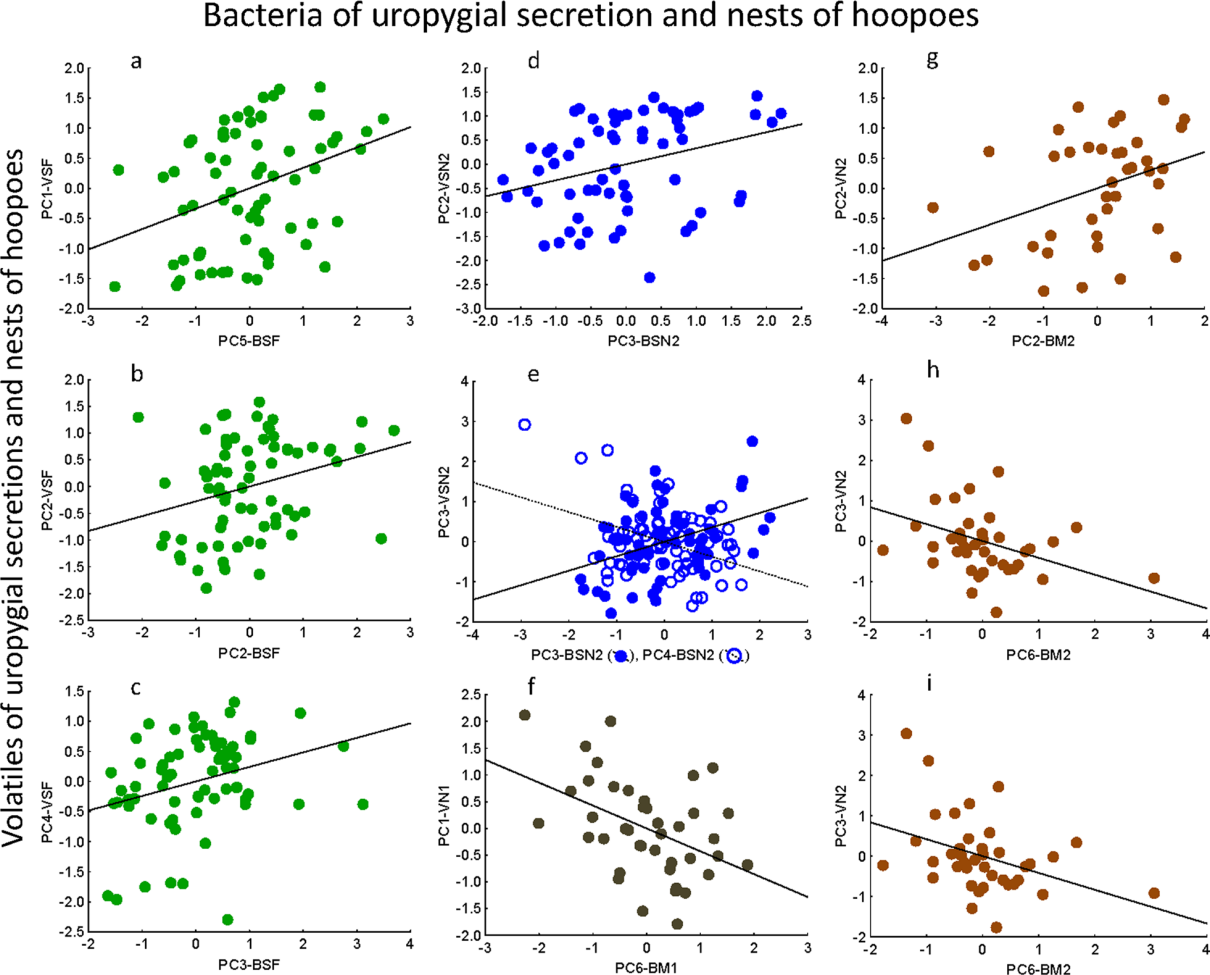
Statistically significant partial associations between scores from Principal Component (PC) axes summarizing relative abundance of detected volatiles (dependent variables) and bacterial (i.e., bacterial genera) (independent factors) of the uropygial secretion of female (green dots: **a**, **b** and **c**) and nestling (blue dots, **d** and **e**) hoopoes, and of their nest materials (bacterial communities) and nest-boxes (volatiles profiles) collected at the beginning (grey dots, **f**) and at the end (brown dots, **g**, **h** and **i**) of the nestling period. Each PC-axis was named by a composition of letters that indicate the type of samples. The first letter indicates whether the sample corresponds to bacteria (B) or volatiles (V), the second letter indicates whether the sample is from secretions of females (SF), secretion of nestlings (SN) or nest material (M). Finally, for distinguishing between types of samples that were collected at the beginning (1) and at the end (2) of the nestling period, the name finished with a number. Lines are regression lines

Relative abundance of particular bacterial taxa and volatiles from the uropygial secretion of adult females and nestlings summarized in PC factors (hereafter PC, Additional file 2: Tables S4 and S5, respectively) resulted related to each other. In adult females, volatile PC1, PC2 and PC4 were related with bacterial PC5, PC2, and PC3, respectively (Fig. 5; Additional file 1: Table S6). In nestlings, at the late stage of their nesting period, volatile PC2 correlated significantly with bacterial PC3, while volatile PC3 associated significantly with bacterial PC2 and PC3 (Fig. 5; Additional file 1: Table S6).

At the beginning of the nestling stage, only bacterial of nest material PC6 explained significantly volatile PC1 of the nest environment (Fig. 5; Additional file 1: Table S6). At the end of the nestling stage, volatiles PC2 and PC4 of the nest environment associated with bacterial PC2 and PC5, respectively (Fig. 5; Additional file 1: Table S6).

### Associations between volatile profiles of nest environments and of secretions

At the beginning of the nestling stage, volatile profiles of the uropygial secretion of nestlings (Mantel tests, R^2^ < 0.017, *p* = 0.047) but not that of adult females (Mantel tests, R^2^ = 0.004, *p* = 0.134) explained the volatile profiles of nest environment.

**Fig. 6:**
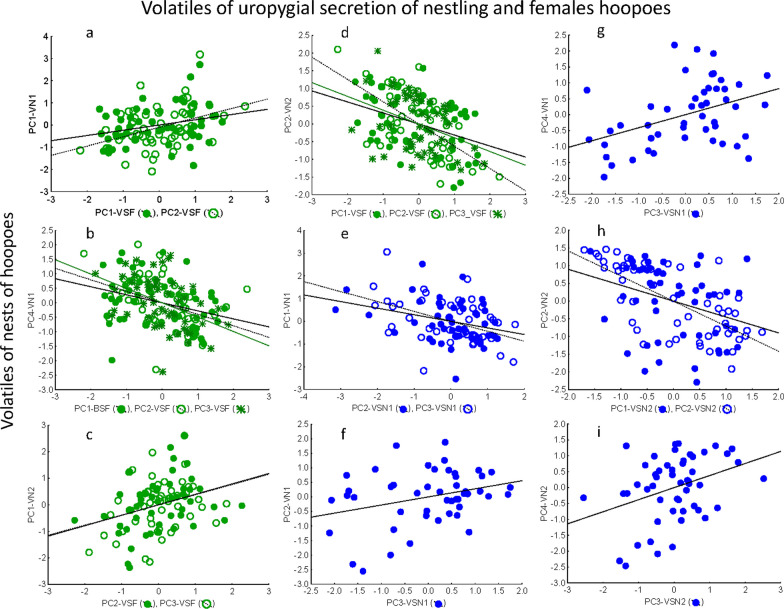
Statistically significant partial associations between scores from Principal Component (PC) axes summarizing relative abundance of detected volatile of hoopoe nest-box environments (dependent variables) and those summarizing volatiles of the uropygial secretion of females (green dots, **a**, **b**, **c** and **d**) and of nestlings (blue dots, **e**, **f**, **g**, **h**, **i**). Each PC-axis was named by a composition of letters that indicate the type of samples. The first letter indicates whether the sample corresponds to bacteria (B) or volatiles (V), the second letter indicates whether the sample is from secretions of females (SF), secretions of nestlings (SN) or nest material (M). Finally, for distinguishing between types of samples that were collected at the beginning (1) and at the end (2) of the nestling period, the name finished with a number. Lines are regression lines

Different volatiles of adult females and nestling secretions associated with the volatile profile of the nest environment (Additional file 1: Table S8). At the beginning of the nestling period, relative abundance of volatiles from female secretion summarized by PC1 and PC2, and by PC1, PC2 and PC3) respectively associated with relative abundance of volatiles in nest environment summarized by PC1 and PC4 (Fig. 6; Additional file 1: Table S8). Similarly, relative abundance of volatiles of nestling secretions (PC2 and PC3) associated with volatile profile of nest environments (PC1, PC2 and PC4) (Fig. 6; Additional file 1: Table S8).

At the end of the nestling stage, volatile profiles of the uropygial secretion of nestlings did not explain the volatile profiles of nest environment (Mantel tests, R^2^ < 0.001, *p* = 0.300). Relative abundance of volatiles in the hoopoe nest-boxes summarized by PC1 related with PC2 and PC3 of adult female volatiles. Moreover, abundance of nest-box volatiles summarized in PC4 associated with that of nestling secretion in PC3 (Fig. 6; Additional file 1: Table S8).

Different set of volatiles showed different scores in these PC axes (Additional file 1: Table S5 and S9). In particular, some aldehydes (pentanal, hexanal and octanal) contributed positively with PC2 of volatiles of nest environment, while acetic and butanoic acids contributed negatively. Hexanal and octanal contributed positively in adult female volatile PC1 and nestling volatile PC1, while some esters contributed in PC2 of both adult females and nestlings.

### Bacteria and volatile of nest environments affecting the intensity of parasitism

**Fig. 7:**
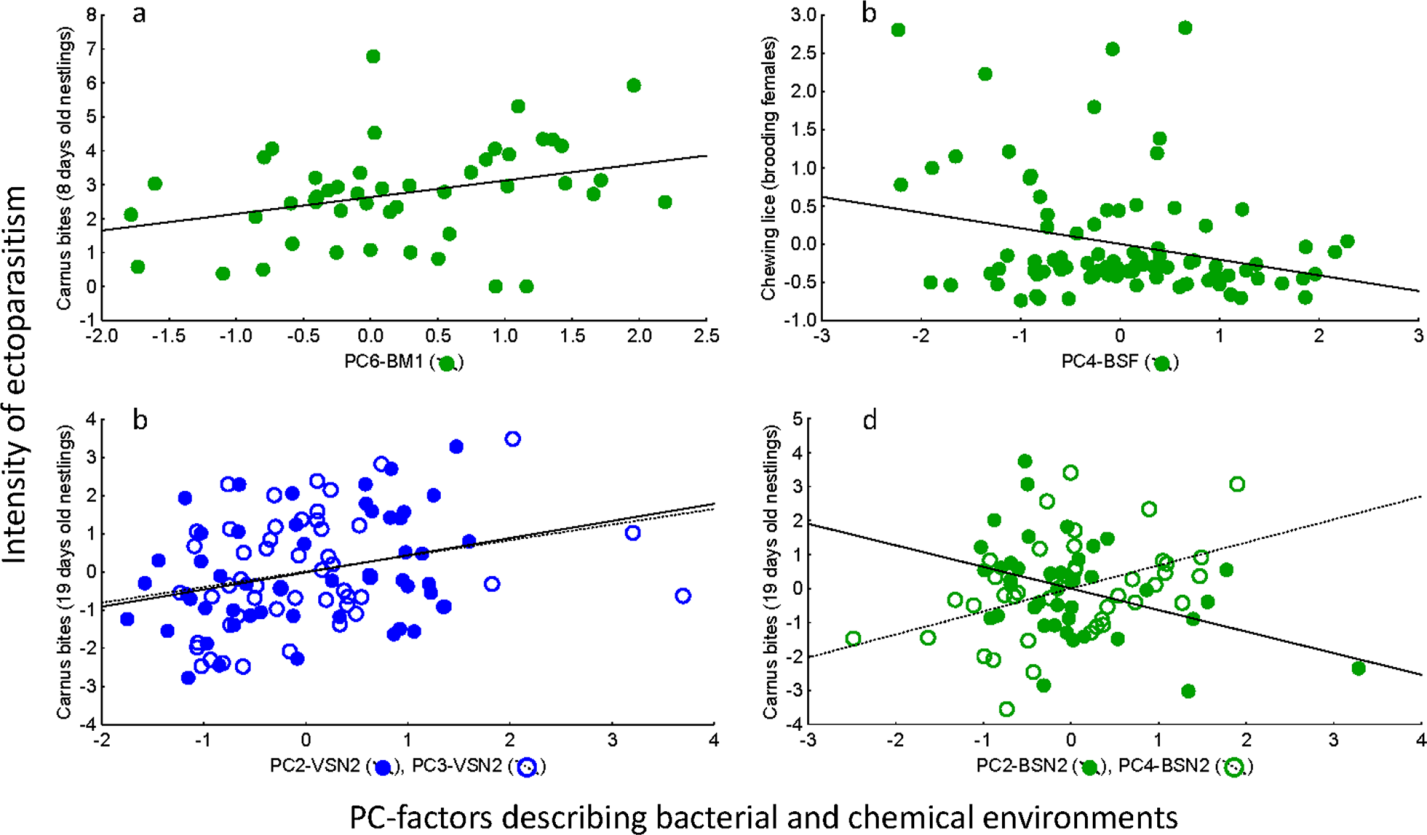
Statistically significant partial associations between intensity of parasitism (number of *Carnus* flies traces) in eight (**a**) and 19 (**b** and **d**) days old nestlings, and females (number of chewing lice) (**c**) (dependent variables) and Principal-Components (PC) scores summarizing relative abundances of detected bacteria (green dots, **a**, **c** and **d**) or volatiles (blue dots, **b**) estimated from samples collected during the early (**a** and **c**) and late (**b** and **d**) nestling periods. Each PC-axis was named by a composition of letters that indicate the type of samples. The first letter indicates whether the sample corresponds to bacteria (B) or volatiles (V), the second letter indicates whether the sample is from secretions of females (SF), secretion of nestlings (SN) or nest material (M). Finally, for distinguishing types of samples that were collected at the beginning (1) and at the end (2) of the nestling period, the name finished with a number. Lines are regression lines

Different PC axes summarizing bacterial community and volatile profile relative abundances significantly associated with intensity of parasitism of 8-days-old nestlings (see Multiple-R values in Table 4 and in Fig. 7). Parasitism was negatively associated with the abundance of some genera belonging to phyla Actinobacteria, Chloroflexi, and Proteobacteria in the nest (PC5-BM1 in Fig. 7; Additional file 1: Table S10).

**Table 4:** Results from GRMs looking for best models explaining parasitism by *Carnus* flies in 8 and 19 days old nestlings, and by chewing lice in females

BACTERIAL COMMUNITY						VOLATILE PROFILES					
	Beta	(SE)	F	df	p		Beta	(SE)	F	df	p
*Parasitism of 8 days old nestlings*											
***Multiple R* = *0.32, F* = *5.45, df* = *1.47, p* = *0.024***						*Multiple R* = *0.20, F* = *2.24, df* = *1,53, p* = *0.141*					
**PC6-BM1**	**0.32**	**0.19**	**5.45**	**1,47**	**0.024**	PC1-VSF	0.20	0.13	2.24	1.53	0.141
*Parasitism of 19 days old nestlings*											
***Multiple R* = *0.68, F* = *7.73, df* = *4,36, p* = *0.0001***						***Multiple R* = *0.40, F* = *4.59, df* = *2,48, p* = *0.015***					
PC6-BM2	**− **0.23	0.13	3.06	1,36	0.089	**PC2-VM2**			**4.87**	**1,48**	**0.032**
**PC2-BSN2**	**− 0.34**	**0.13**	**6.40**	**1,36**	**0.16**	**PC3-VM2**			**4.12**	**1,48**	**0.048**
**PC4-BSN2**	**0.36**	**0.13**	**8.47**	**1,36**	**0.006**						
PC6-BSN2	0.24	0.12	3.69	1,36	0.062						
*Parasitism of adult females*											
***Multiple R* = *0.3, F* = *4.24, df* = *3,88, p* = *0.008***						*Multiple R* = *0.12, F* = *1.32, df* = *1,94, p* = *0.254*					
PC2-BFS	0.19	0.10	3.71	1,88	0.057	PC4-VSF	**− **0.12	0.10	1.32	1,94	0.254
**PC4-BFS**	**− 0.26**	**0.10**	**6.88**	**1,88**	**0.010**						
PC5-BFS	0.15	0.10	2.12	1,88	0.149						
*Fledging success and variables from the early nestling period*											
*Multiple R* = *0.42, F* = *2.78, df* = *3.40, p* = *0.054*						***Multiple R* = *0.36, F* = *4.72, df* = *2,62, p* = *0.012***					
PC2-BM1	0.23	0.15	2.42	1,40	0.128	PC4-VN1	**− **0.26	0.15	2.74	1,36	0.107
PC3-BM1	**− **0.23	0.15	2.48	1,40	0.124	PC3-VSF	**− **0.27	0.14	3.69	1,36	0.063
PC5-BSF	0.27	0.15	3.22	1,40	0.080	**PC1-VSN1**	**− 0.48**	**0.13**	**13.72**	1,36	**0.001**
						**PC2-VSN1**	**0.34**	**0.14**	**6.16**	**1,36**	**0.018**
						**PC3-VSN1**	**0.35**	**0.15**	**5.23**	**1,36**	**0.028**
*Fledging success and variables from the late nestling period*											
***Multiple R* = *0.59, F* = *3.72, df* = *5,35, p* = *0.008***						*Multiple R* = *0.31, F* = *2.47, df* = *2,48, p* = *0.096*					
**PC3-BM2**	**0.34**	**0.15**	**5.14**	**1,35**	**0.030**	PC2-VSN2	-0.23	0.14	2.83	1,48	0.100
PC6-BM2	0.26	0.15	3.05	1,35	0.090	PC4-VSN2	0.22	0.14	2.61	1,48	0.113
**PC1-BSN2**	**− 0.32**	**0.14**	**5.27**	**1,35**	**0.028**						
PC3-BSN2	0.24	0.16	2.41	1,35	0.130						
**PC5-BSN2**	**− 0.36**	**0.16**	**4.82**	**1,35**	**0.035**						

Best models were separately analysed for bacterial (B) and volatiles (V) PCs. As potential variables to explain parasitism of nestlings in booth ages we considered those from the nest material and environment (M) and from the uropygial secretion (S) of females (F) and nestlings (N) collected at the beginning (1) and at the end (2) of the nestling period, respectively. We show multiple R of the best models and highlight partial effects with *p*-values lower than 0.05 in bold font

Bacterial and volatile profiles of nestling secretions also explained intensity of parasitism of nestlings close to fledge (see Multiple-R values in Table 4 and Fig. 7). PC2 and PC4 axes summarizing abundance of particular bacteria of nestling secretions related negatively and positively, respectively, with nestling parasitism by *C. hemapterus* (Fig. 7; Table 4). Bacterial genera belonging to the Order Clostridiales showed the highest and the lowest scores in these axes, indicating their importance in nestling parasitism (Additional file 2: Table S4 and Additional file 1:Table S10). In regards with volatiles, abundance of those summarized by PC2 and PC3 axes of the nest environment explained intensity of parasitism of nestlings at this late stage (Fig. 7; Table 4). In these axes, aldehydes are the volatiles that most contributed (Additional file 2: Table S4 and Additional file 1: Table S10).

Finally, intensity of chewing lice parasitism of adult females only associated with PC4 of microbiota of female secretions (Fig. 7; Table 4). Again, genera of the Order Clostridiales mostly contributed positively and negatively to this axis (Additional file 2: Table S4 and Additional file 1: Table S10).

For a detailed description of bacterial genera and volatiles summarized in different PC-axes see Additional file 2: Tables S4, S5 and Additional file 1: Table S10.

### Fledging success and bacterial and volatile profiles of hoopoe nests

**Fig. 8:**
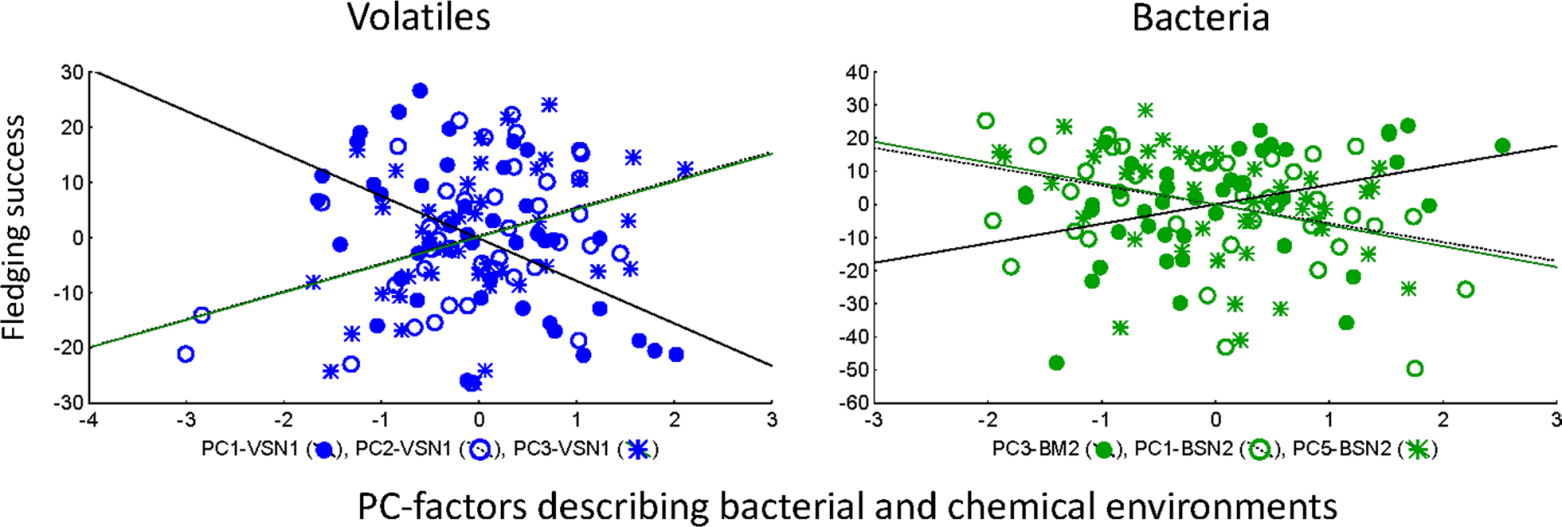
Statistically significant partial associations between fledging success (dependent variable) and Principal-Components (PC) scores summarizing relative abundances of detected bacteria (green dots) in the nest material (PC3_BM2) and in the secretion of nestlings (PC1-BSN2 and PC5-BSN2) at the late stage of the nestling period; and between fledgling success and relative abundance of detected volatiles (blue dots) of the secretion of 8 days old nestlings (PC1-VS1, PC2-VS1, PC3-VS1). Lines are regression lines

Only PC axes summarizing relative abundance of particular volatiles of nestling secretion at early states of the nestling period explained fledging success (PC1, PC2 and PC3; Fig. 8, Table 4). In this case, several esters and acids were those that mostly contributed to these axes (Additional file 2: Tables S4 and Additional file 1: Table S10).

At late stage of the nestling period, PC3 summarizing abundance of bacteria of the nest material, as well as PC1 and PC5 summarizing bacterial abundance of nestling secretions correlated significantly with fledging success (Fig. 8, Table 4). In spite of the diverse genera that contributed to these axes, most of them were genera belonging to the Order Clostridiales (Additional file 2: Table S4 and Additional file 1: Table S10).

For a detailed description of bacterial genera and volatiles summarized in different PC axes, see Additional file 2: Tables S4 and S5 and Additional file 1: Table S10.

## Discussion

As far as we know, this is the first study exploring the hypothetical link between bacterial symbionts, animal odours and ectoparasitism within the same study system. By manipulating bacterial communities of hoopoe nests before reproduction started, we detected effects on different components of the bacterial communities and volatile profiles of nests and bird uropygial secretions during the nestling phase, and on intensity of parasitism in nestlings and adult females. Volatile profiles of the nest environment associated with those of secretions at the beginning of the nestling phase and relative abundance of particular volatiles and bacteria of hoopoe nests and nestlings and adult female secretions summarized by PC axes predicted the intensity of ectoparasitism suffered by brooding females and nestlings. All these results are in accordance with the hypothesis that bacterial communities are partially responsible for the volatile profile of avian nests, which ultimately affect risk of parasitism and fledging success. Below, we discuss the importance of the detected associations in light of previous results and of the hypothesis tested and alternative explanations.

Previous work in the same study system and hoopoe population demonstrated that the presence of unaltered nest material from previous hoopoe breeding attempts increased bacterial density on the eggshells [54], and in the nest materials during the nestling phase [45]. In the present work, we have shown that bacterial communities of nest material in experimental nests (autoclaved) during the nestling phase were less diverse and clustered apart from those of control nests, confirming that bacterial communities before reproduction determine the bacterial environment during the nestling phase. Interestingly, we have detected parallel experimental effects on bacterial communities and on the chemical environment of nests during the nestling phase, with experimental nests showing more diverse volatile profiles than control nests. Moreover, characteristics of symbiotic bacterial communities of the uropygial secretion of adult females and nestlings associated with particularities of their volatile profiles. These results add experimental and correlational support to the expected association between bacterial communities and volatile profiles of birds, which was previously demonstrated by injecting antibiotics in the uropygial gland of hoopoes [25] and dark-eyed juncos (*Junco hyemalis*) [28].

Our hypothesis posits that bacterial symbionts in their uropygial glands and the microbial communities in their nests are partially responsible for the general nest odour that conveys inadvertent social information to ectoparasites. The hypothesis therefore assumes that bacterial and volatiles components of avian nests environment, including those of birds, should be related to each other, and our results support the assumption. Volatile profiles of the nest material at the end of the nestling period associated with the profile of volatiles captured inside the hoopoe nest boxes. Moreover, PC-scores reflecting relative abundance of particular volatiles in the nest-box and secretion of adult females and nestlings, respectively, correlated with PC-scores summarising relative abundance of bacteria of the nest environment and secretion of females and nestlings. Although causality cannot be inferred from these correlative results, the detected parallel effects autoclaving nest materials on both bacterial communities and volatiles profiles of hoopoe nest environments, suggest that bacteria causing volatile characteristics of hoopoe nests is the most likely direction of detected associations.

Our hypothesis also suggest that females and nestling odours, which mainly come from their uropygial secretions, should be partially responsible of nest odour. In accordance, volatile profiles of the uropygial secretion of nestlings explain a reduced, but statistically significant, proportion of variance of the volatile profiles of hoopoe nests at the beginning of the nestling period. Moreover, several PC-scores summarising relative abundances of volatiles in the uropygial secretions of females and nestlings associated to those in the nest environment. Uropygial secretions were collected from the inside of the uropygial gland and, thus, it is unlikely that volatiles of the nest boxes determine those of the secretion. Consequently, our results indicate that odours of hoopoe nests are partially determined by uropygial secretion odours and, because odours associate with bacterial communities and both odours and bacterial communities were affected by bacterial clearance of nest materials, all those results considered together suggest that bacterial communities determines the odour of hoopoe nests. A previous work demonstrated that eliminating bacteria in the preen gland using an antibiotic produced changes in volatiles of secretions [25], so our findings further support the hypothesis that symbiotic bacteria of the preen gland contribute to avian nest odour.

Our hypothesis states that parasites use volatiles of bacterial origin to detect and/or choose the nests of their hosts [9]. In a previous paper with identical set of nests, we showed that nestlings grown in nest-boxes with experimentally autoclaved nest materials suffered lower intensity of ectoparasitism by *Carnus* flies than those of control nests, while bacterial loads of nest material associated positively with fledging success [45]. In that paper, we suggested that volatiles from bacteria metabolism could be responsible for the detected experimental effects on intensity of parasitism. That suggestion was based on previous results demonstrating the links (i) between autoclaving the nest material and characteristics of bacterial communities of the uropygial secretions of adult females and nestlings [54], and (ii) between symbiotic bacteria and the volatiles of the uropygial secretion of hoopoe nestlings [25]. Here, using information from high-throughput sequencing of bacterial communities and GC–MS chemical analyses of uropygial secretions of nestlings and adult females, and of nest-environment, we were able to test and find further support of a possible role of bacterial partially determining volatiles of hoopoe nests (see above). In relation to parasitism, we found that scores of PC-axes reflecting relative abundance of particular bacterial groups from nest material, as well as particular volatiles of the secretion of nestlings and of nest environment, predicted intensity of parasitism of young nestlings by *Carnus* flies. Similarly, PC-scores reflecting abundance of particular bacteria from nest materials collected at the end of the nestling period, as well as volatiles of the uropygial secretion of close-to-fledge nestlings, associated with their intensity of parasitism. Furthermore, abundance of chewing lice on adult female feathers associated with particular bacteria and volatiles of their uropygial secretion. Therefore, independently of the identity of bacteria or chemical component responsible of the detected associations with parasitism, our results support the hypothetical role of symbiotic bacteria, and of their volatiles on the interactions between hoopoes and their ectoparasites. Importantly, since we found support to the hypothetical role of bacteria partially determining nest odour, it is likely that the detected associations between parasitism and bacteria or volatiles were primary explaining by bacteria partially determining chemicals that parasites use to detect and select host nests for parasitism.

In any case, correlations do not imply causality and, thus, associations between parasites and bacteria or volatiles might be interpreted in both directions: volatiles produced by bacteria affect parasitism; or parasites affect bacterial communities and volatiles of their hosts. Parasites, by definition, use host resources for their own and thus influence physical condition and health of their hosts [59]. Since host physical or physiological condition influence characteristics of their microbial symbionts and vice versa [3], parasitism could affect their microbial symbiotic community [reviewed in^9^, ^60^–^62^]. Nutritional condition of hosts also affects their immunological resistance to parasites [55, 59, 61]. Thus, the effects of parasitism on nutritional condition and immune competence of their hosts could indirectly explain the detected association between bacteria and parasitism. Given the complexity of the interacting mechanisms potentially explaining associations between parasitism, immunity, and symbiotic bacterial communities of animals [3], the manipulation of bacterial communities is essential to demonstrate their effects on parasitism. Indeed, we manipulated bacterial communities of nests before reproduction, which affected not only parasitism intensity but also volatile profiles and bacterial communities of the hoopoe nests during the nestling phase. Then, the most likely explanation for the detected associations between particular groups of volatiles and of bacteria with intensity of parasitism is that volatile-producing bacteria affects parasitism, and not the reverse. Experimental manipulations of these components (volatiles, bacteria, or parasitism) during the nesting phase are however need to reach firm conclusions.

Relative abundance of some aldehydes, acids and esters associated with intensity of parasitism in nestlings and adult females, and with fledging success. Most of these chemicals have been detected in other animals and are known as determining host selection by some arthropod pests [40]. Nonanal, for instance, is a typical component of the volatile profile of some birds (i.e. pigeons and chickens) that attracts mosquitoes of the genus *Culex* [63], and it has been used as sentinel in sighting pest programs [64–66]. Similarly, relative abundance of different bacterial genera belonging to order Clostridiales in the uropygial secretions associated positively and negatively with parasitism intensity and fledging success. We have shown previously that genera of this bacterial Order are relatively highly abundant in uropygial secretions of hoopoes [52, 53]. Moreover, taxa belonging to the Order Clostridiales are common bacteria in bird guts [67]. Consequently, these taxa could play an important role that deserves future research to untangle their role in parasite-host communication.

## Conclusions

In general, results presented here support the links between microbial communities and animal odours, and emphasize that the associations between symbiotic bacteria and both ectoparasitism and reproductive success are partially mediated by volatiles that may be of bacterial origin. Future work should focus on mechanisms underlying the detected patterns.

The detected associations are in any case complex and our results strongly support a central role of volatiles of symbiotic bacterial origin. However, the importance of different bacterial taxa and of different volatile compounds determining risk of parasitism and fledging success urges further experimental approaches.

These associations between some groups of bacteria and volatiles of different types of samples associated with fledging success indicating that these chemical and microbiological components may influence host fitness.

## Material and methods

### Study area and species

The study area was located in the Hoya de Guadix, (Granada, Southern Spain, 37°18′N, 38°11′W), a plateau at 1000 m a.s.l. with semiarid climate, where around 300 cork-made nest-boxes were available for wild birds; most of them attached to tree trunks and walls, but also hidden in piled stones. The dimensions of nest-boxes were 35 × 18 × 21 cm (internal height × width × depth), 24 cm (bottom-to-hole height) and 5.5 cm (entrance diameter). Information about the study area is further described elsewhere [54, 68].

The hoopoe (Order Upupiformes) is a migratory species, distributed throughout Europe, Asia, and Africa [69–71]. Hoopoes are hole-nesters that frequently use artificial nest-boxes for reproduction or natural cavities in trees or walls. They do not build nests and prefer cavities with remains of soft material from previous reproductive events of conspecifics or heterospecifics [54], where adult females create a small hollow and lay the clutch [56]. In our study area, the reproductive season starts in late February, and extends until late July.

*C. hemapterus*, a common generalist hematophagous parasitic fly of about 2 mm in length [57, 72] usually cause bleeding at the sucking spots. In hoopoes, chewing lice are common on the feathers of the crest where they are more protected from bird preening, as birds can only reach this area with their feet. Chewing lice feed by chewing soft areas of the feathers and skin of their avian hosts [73, 74].

### Fieldwork and experimental design

Fieldwork and labwork, including autoclaving and sampling collection, were carried out during 2017 and 2018. Before reproduction started (i.e. beginning of February), we visited nest-boxes where hoopoes successfully bred the previous year. We collected old nest materials (47 nest-boxes in 2017 and 70 in 2018) in plastic bags perforated with small holes to maintain the nest material in aerobic conditions. Nest materials were stored at room temperature in the lab for three weeks until it was used to fill new installed nest-boxes. Hence, nest materials from different nest-boxes were pooled, mixed, and divided in two halves. *C. hemapterus* flies pupate overwinter inside bird nests, and autoclaving the nest material should kill them. Therefore, after treatment, experimental and control material could differ not only in bacterial density but also in the probability of *C. hemapterus* flies emerging within the control nest boxes, which might affect experimental outcomes. To evaluate this potential bias, we removed in 2018 all pupae from the material of both experimental treatments before autoclaving. We did so by sifting nest-material with opening meshes of 2, 1, and 0.5 cm diameters. Interestingly, it was in that year when the expected effect of experimental autoclaving nest material on intensity of parasitism was clearer [45]. Moreover, to avoid any possible influence of previous reproduction on bacteria communities and volatile profiles, we installed new nest-boxes (86 in 2017 and 69 in 2018). Most of the times, new nest-boxes substituted old ones, although we placed also new nest-boxes in new locations in the field with any of the experimental levels. New nest-boxes were sequentially assigned to one of the two experimental treatments, adding 500 cm^3^ of nest material from previous reproduction, that were (experimental) or were not autoclaved (control), mixed with 500 cm^3^ of commercial sawdust (Allspan® Animal bedding, wood shavings). This procedure was performed by wearing new pair of latex gloves cleaned with 96% ethanol for each nest-box to avoid cross-contamination between experimental and control nests materials. The third group of nest-boxes (i.e., natural nests), old nest-boxes were neither new nor manipulated, and where hoopoes bred the year of sampling (N = 13 in 2017 and N = 14 in 2018). As bacteria and volatile environment likely depends on previous reproduction, we here only considered first breeding attempts.

During reproduction, from early March to the end of June, both new (i.e., experimental and control) and old (i.e. natural) nest-boxes in the study area were visited every four days until eggs were found inside. Hoopoes lay one egg per day, and clutch size is typically of seven eggs, start incubation with the first or second egg and incubation last around 17 days [75]. Then, the rate of nest visiting allowed us to estimate laying date (i.e. that of the first egg). Nest-boxes where inspected again 17 days after the onset of egg laying, and daily afterwards until detecting hatching date (day 1 of the nestling period), which is expected to occur 17 days after the onset of incubation [75]. During the nestling stage, nests were visited seven times (details below and Additional file 1: Table S1) to sample nest materials, volatiles of the nest environment and uropygial secretions, as well as to record parasitism intensity of adult females and nestlings (Additional file 1: Table S1). For collecting each of these samples within a nest, we wore new pair of latex gloves previously cleaned with 96% ethanol to avoid contamination between samples. Multiple visits were needed to ensure that disturbances to the nests due to the time required to collect samples were not unduly long (i.e. multiple visits needed to minimize negative impacts of researcher visits that could have biased breeding success).

### Bacterial, volatile and ectoparasite sampling

For bacterial community analyses, approximately 10 g of the nest material in contact with nestlings was collected twice in 15 mL Falcon tubes, on days 4 and 15 after the first egg hatched (Additional file 1: Table S1). These samples were stored in a portable fridge until being frozen at – 20 °C in the laboratory within the same day of collection.

Volatile compounds of the nest environment were also sampled twice, on days 7 and 18 after the first egg hatched (Additional file 1: Table S1). Volatiles were captured in Solid Phase Microextraction (SPME) fibres. Each fibre was installed at one side of the nest-box, about 7 cm over the nest material, while the sensitive fibre end was protected with a two-side opened glass pipette tip and exposed to the nest environment for 24 h. Afterwards, the fibre was removed from the nest and the fibre end introduced into a sealed glass vial, kept cold (0–4 °C), and stored within the same day at – 20 °C until gas chromatography–mass spectrometry analysis. Storage of samples never exceeded one week. After the analyses, SPME fibres were re-conditioned (i.e., all chemical trace eliminated) following supplier instructions, i.e. 1 h at 270 °C using the GC injector and afterwards, fibres were kept at – 20 °C until they were reused in the field.

Uropygial secretions were collected on days 4 (adult females), 8 and 19 (nestlings) after the first egg hatched (Additional file 1: Table S1). Briefly, before sampling, the uropygial gland and surroundings were cleaned with a cotton swab soaked in 96% ethanol. Afterwards, we used an automatic 1–10 μL micropipette and gently introduced the sterilized tip into the papilla of the uropygial gland and pipetted the secretion. At least 5μL of secretion from adult females or close-to-fledge nestlings was placed in a sterile 1.5 mL microcentrifuge tube for bacterial DNA analyses. In addition, 10 μL were transferred to 10 ml SPME sealed glass vials for analyses of the volatile profile. Six to eight days old nestlings produce scarce uropygial secretion, so these secretions were employed only for exploring volatile profiles of nestlings. Since we were interested on the chemical profile of nests, we sampled a single nestling per nest (usually the largest one) at this nestling stage, and, when not reaching 10 μL volume, we completed with the secretion of other siblings. At the end of the nestling stage, we sampled all nestlings in the nest: Following same philosophy, we collapsed volatile and bacterial community data by nest (see below). Samples were kept cold in a portable fridge and then stored at – 20 °C within a week for chemical volatile analyses or until DNA extraction.

Ectoparasitism of adult females was estimated on day 4 after the first egg hatched by counting the number of chewing lice on the crest feathers, which is a good proxy of parasitism intensity [73, 74]. Ectoparasitism of nestlings was estimated twice, when the older nestling was 8 and 19 days old (Additional file 1: Table S1). These estimates consisted of counting the number of spots due to faeces and blood remains on the belly skin and left underwing of all nestlings. These spots are traces of *C. hemapterus* parasitism and reflect the abundance of ectoparasites and, thus, the intensity of parasitism of nestlings [76, 77]. We used mean values per nest in the analyses. Finally, fledging success was estimated as the percentage of nestlings that survived from day 8 after hatching of the first egg until day 19.

### DNA extraction and high-throughput sequencing

Bacterial DNA from secretions and nest material were extracted using the FavorPrep Blood Genomic DNA Extraction Kit (Favorgen Biotech) and MSOP protocol [78], respectively. For secretions, and according to manufacturer’s instructions, we added a lysozyme pre-treatment (10 mg/mL of lysozyme at 37 °C for 30 min) to ensure the DNA extraction of Gram positive bacteria [53]. Nest material samples were solid and 80 mg were used for DNA extraction. First, samples were suspended with 900 µL of lysis buffer. The liquid phase was then separated from the solid content and kept in different 2 mL microfuge tubes (for further details of the followed protocol see Lee et al. 2021). Extracted DNA from nest material samples was cleaned using the kit One Step PCR Inhibitor Removal Kit (Zymo Research). We also processed laboratory blanks to detect possible contamination during the process.

DNA sequences from nest materials and uropygial secretions were obtained by Illumina high-throughput sequencing of a fragment of approximately 400 bp of the 16S rRNA V6-V8 hypervariable regions. In a first PCR, 16S rRNA gene were amplified using universal primers B969F (5′-ACGGGCRGTGWGTRCAA-3′) and BA1406R (5′-ACGGGCRGTGWGTRCAA-3′) [79]. PCR was carried out at final volume of 25 µL containing 12.5 µL of iProof High-Fidelity DNA Polymerase (Bio-Rad Laboratories, Inc.), 0.3 µM of each primer, and 5 µL of template DNA. PCR conditions included an initial denaturing step of 98 C for 1 min followed by an amplification step of 25 cycles of 10 s at 98 C, 20 s at 52 C, and 15 s at 72 C, and a final extension of 5 min at 72 C. In a second PCR, samples were amplified adding barcodes for identifying samples. PCR was carried out at final volume of 25 µL containing 12.5 µL of iProof High-Fidelity DNA Polymerase (Bio-Rad Laboratories, Inc.), 0.3 µM of each primer, and 5 µL of template DNA. PCR conditions included an initial denaturing step of 98 C for 1 min followed by an amplification step of 25 cycles of 10 s at 98 C, 20 s at 52 C, and 15 s at 72 C, and a final extension of 5 min at 72 C. Afterwards, the libraries were sequenced in a single run of Illumina MiSeq sequencer (2 × 300 bp output mode). Sequencing was carried out at the Integrated Microbiome Resource (IMR), University of Dalhousie (Canada). Sequences are available at NCBI under accession numbers: BioProyect ID: PRJNA847390 (nest samples) and BioProyect ID: PRJNA847428 (secretion samples).

Raw sequences were analysed using QIIME2 2019.10 [80]. Briefly, primers were trimmed and, due to low quality of the reverse sequences, analyses were based on forward sequences. Sequences were quality filtered following default parameters in QIIME2 and the Deblur algorithm was employed to produce an Amplicon Sequence Variants table (ASV table) [81], with a sequence size of 220 bp.

Afterwards, a phylogenetic tree was built using the fragment insertion algorithm (Janssen et al. 2018). Taxonomic assignation was performed against Greengenes13_8 database at 97% similarity [16, 82]. Chloroplast, mitochondria and non-phylum assigned ASVs were removed.

Sequences with fewer than 0.1% of reads and fewer of 10% of samples were filtered out and samples with fewer of 5000 sequences were removed from the ASV tables following criteria of Grieves et al*.* [27, 34]. Since we were interested on the chemical profile and bacterial community of nests, we collapsed tables related with nestlings at the late nestling period by calculating average values of volatiles and ASV by nests using QIIME2. Due to zero counts could mask true zeros from too low counts for Illumina detection, we replaced those zero values using a Bayesian-multiplicative replacement to impute values for zero count sequences. For this purpose, we used the R package zCompositions [83]. Afterwards, we applied a centred log-ratio (clr) transformation to the ASV tables [84, 85].

### Volatile profiles: gas chromatography-mass spectrometry (GC–MS)

**Fig. 9:**
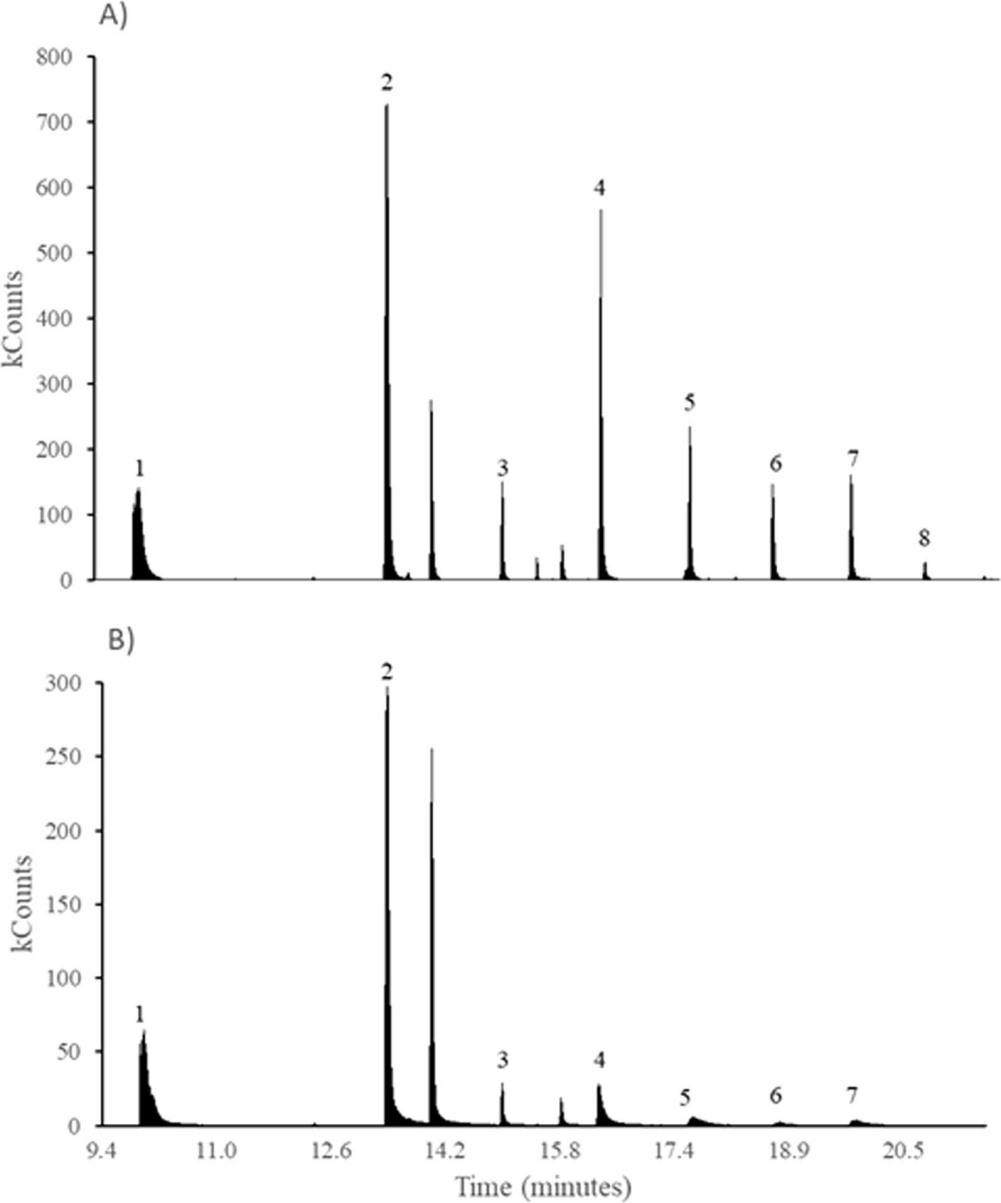
Example of SIM 60 m/z chromatograms of **A**) the nest environment of a hoopoe nest at the beginning of the nestling stage and of **B**) uropygial secretion of the brooding female. Peaks represent the acids detected in kcounts (from fewer carbons to higher carbons in the molecule: 1. acetic, 2. butanoic, 3. pentanoic, 4. hexanoic, 5. heptanoic, 6. octanoic, 7. nonanoic and 8. decanoic)

Analyses were performed on a gas chromatograph coupled to a mass spectrometer Varian 450GC 240MS with an automatic injector Combi Pal to SPME fibre (50/30 μm DVB/CAR/PDMS, Stableflex 23 Ga, Autosampler). Injector desorption was performed at 250 °C for 10 min in Split (20: 1) and helium flow at 2 mL/min. The capillary column was Aligent HP-FFAP 30 m × 0.32 mm × 0.25 μm. The oven temperature was initiated at 50 °C for 1 min, and programmed to increase 5 °C /min to 100 °C, then at 10 °C /min to 200 °C and 50 °C /min to 250 °C for 1 min. The scan range of the mass spectrometer was in mode TIC Full Scan between 30 and 500 m/z. The identification of compounds was established by characteristic ion SIM analysis and the NIST 08 spectrum library (Fig. 9). The summary of volatile profiles of next-box environments and uropygial gland secretions is in the Electronic Supplementary Material (Additional file 1: Table S7). Standards of pure compounds were used when necessary for confirmation.

Volatile profiles were calculated as the relative abundance of each volatile in the chromatogram, i.e. the percentage of the area of each compound over the total area of the chromatogram adjusted to 100%. The number of volatile samples collected from experimental, control and natural nest-boxes is listed in Additional file 1: Table S1.

### Statistical procedures

Alpha diversity analyses (i.e. the microbial diversity within a particular sample) [86], of bacterial communities was estimated with the ASV table before zero replacement as by means of Shannon and Faith’s phylogenetic diversity (Faith’s PD) indexes in QIIME2 [80]. For beta diversity analyses (i.e., the variability in community composition among different samples) of bacterial samples [86], we calculated Aitchison distance, that calculates the Euclidean distance between pair of samples [87] and PhilR, which take into account the phylogenetic relationships among bacterial taxa [88].

For volatiles of the nest environment and uropygial gland secretions, we estimated alpha diversity using Shannon index, and beta diversity using the Aitchison distance between samples [87].

Alpha diversity indexes and Aitchison calculations were calculated using the default R package *stats*, while the *philr* package [88] was employed for the generation of the PhilR distance matrixes. This packages were implemented in R.3.6.1 [89].

General linear models (GLM) were performed to study the effect of experimental treatments on alpha diversity estimates. Distribution of alpha diversity values did not differ significantly from a Gaussian distribution (Kolmogorov–Smirnov test for continuous variables, *P* > 0.05). Full models included treatment and study year as fixed factors, laying date as covariable and interactions between treatments and study year. In case of interactions did not reach statistical significance, we run models without these terms. Residuals of all GLMs approximately followed normal distributions.

The effect of treatment on the beta diversity of bacterial communities and of volatile profiles were explored by means of PERMANOVAs (i.e., nonparametric multivariate analysis of variance) with 10 000 permutations. Full models included treatment and study year and early (end of March, April and first half of May) versus late (second half of May and June) reproduction as fixed factors and interactions between fixed factors. In case of interactions were not significant, we run models without these terms. These analyses were performed in PRIMER-7.0.17. To visualize segregation of bacterial communities and of volatile profiles due to experimental treatment, we performed Principal Coordinates Analyses (PCoA) plots, implemented in Emperor 2018.2.0 [90]. Finally, we used Analyses of Composition of Microbiomes (ANCOM) [91], implemented in QIIME2, to determine which particular bacterial genus, or particular volatiles are responsible for the detected differences among experimental, control and natural nests.

The associations between bacterial and volatile profiles of different types of samples were explored by mean of Mantel-test with 9999 permutations using the function MRM in the *ecodist* package [92] as implemented in R.3.6.1 [89]. That statistical test is equivalent to a multiple regression but using matrices of differences among samples in a multivariate setting. Importantly, given that for exploring the associations between bacterial and volatile profiles the matrices have to be estimated with the same methodology, we here calculated bacterial matrices based on Bray–Curtis dissimilarities. We selected the same samples in both matrices in QIIME2 before Mantel-test analyses to get the same dimensions in each analysis.

To explore the associations between specific bacteria and specific volatiles of uropygial secretion and nest material samples, we used PC-scores (i.e., after varimax rotation) from Principal Component Analyses (PCA) of the clr-transformed relative abundances of the bacterial taxa (considering the first six axes) and of the clr-transformed relative abundance of the volatile components. Because the former PCA included more variable (i.e. taxa) than the later (chemical), the number of considered PC-axes in subsequent analyses were larger in those belonged to bacterial taxa (first six axes) than in PCAs of detected volatiles (four first axes). The associations between specific PC-scores describing relative abundance of particular volatiles (dependent variables) and those describing bacterial abundance (independent factors) were explored by mean of GLMs. Because we performed multiple GLMs (one per each PC-axis of volatiles), p-values describing the strength of the partial associations were adjusted for the effect of multiple testing [FDR, 93]. Similarly, we performed GLMs to explore the association between PC-scores describing relative abundance of volatiles of nests (dependent variable) and those of the uropygial secretion of adult females or nestlings (independent factors). In this case, associations were separately estimated for adult females and for nestlings, and, thus, we performed eight different GLMs. *P*-values were also adjusted following the FDR procedure [93]. PC-scores were also used to explore the associations between bacteria or volatiles components and intensity of parasitism or fledgling success.

To search for the best combination of PC-axes describing bacterial communities and volatile profiles that explained the parasitism intensity of hoopoe nestlings and adult females, as well as fledging success, we used best subsets General Regression Models (GRM). The best subsets GRM was estimated by means of Mallow’s CP [94], which is equivalent to Akaike information criterion (AIC) [95]. Importantly, information on bacterial and chemical profiles were not always available for the same pool of nests, so we separately analysed the effect of chemical and bacterial information. Moreover, we did not successfully collect information for all types of samples within the same nest (e.g., nestling secretion, adult female secretion or nest material) and, thus, available sample sizes for models that included all types of samples were reduced. When none of the PC-axes describing bacterial (or volatiles) variation of one or more types of sample entered in the best model, we again ran the GRM but excluding these term from the list of independent factors.

GRMs directed to identify best models explaining parasitism intensities estimated at different nest stage (i.e., early vs late nestling stage) included independent factors that summarize information on bacterial communities or volatiles profiles that were collected in appropriate nestling stage. In models that explores factors explaining parasitism of recently hatched hoopoe nestlings and in adult females, initial GRM included information on the bacterial communities or on volatile profiles of nests, nestling and adult female secretions that were collected at the beginning of the nestling period. Similarly, initial GRM explaining intensity of parasitism of nestlings close to fledging included information on bacterial communities and volatile profiles of material collected at the end of the nestling period (i.e., nest material and uropygial secretion of nestlings that were close to fledge). Finally, PC-factors accounting for bacterial and volatiles profiles of the secretions of females were used in the GRM analyses trying to explain chewing lice parasitism of adult females. We did so because we do not expect a major effect of bacteria or bacterial-derived volatiles from the nest or the nestlings on lice attraction in adult females, whose major mode of transfer is supposed to be through direct host contact [96]. GRM models were also used to search for factors explaining fledging success. To this aim, we performed two different GRMs that respectively considered information from samples collected during early or late nestling stages.

Frequency distributions of square root transformed values of the intensity of ectoparasitism in nestlings, as well as raw values of fledging success did not differ significantly from Gaussian distribution (Kolmogorov–Smirnov test for continuous variables, *p* > 0.05). GLMs, GRMs and PCAs were performed in STATISTICA 12 software.

### Supplementary Information

**Additional file 1. Table S1. **Number of experimental (with autoclaved nest material), control (with non-autoclaved nest material) and natural (with old nest material) hoopoe nests with information of bacterial community, volatile profiles and parasitism at different nestling stages (day of sampling: days (d) after the first egg hatched). Bacterial and volatile samples were collected from the nest environment [nest material (Nest Mat) or nest air (Nest-box) and from the uropygial gland (secretion) of females and nestlings. **Table S2.** General Lineal Models exploring the effect of autoclaving nest material on Shannon (Sh) and Faith’s phylogenetic diversity (Pd) indexes of bacterial communities and volatile profiles at different nesting stages and nest locations. We show least square means (SE) for control and experimental (Exp) treatments, and for different study years, and beta (SE) values of the associations with laying date. Statistical effects lower than 0.05 are in bold. **Table S3.** General Lineal Models exploring the effect of using new nest boxes (i.e., Natural vs Control) on Shannon (Sh) and (Pd) alpha diversity indexes of bacterial communities and volatile profiles at different nesting stages and nest locations. We show least square means (SE) for control and experimental (Exp) treatments, and for different study years, and beta (SE) values of the associations with laying date. Statistical effects lower than 0.05 are in bold. **Table S6.** Multiple regression exploring the effect of the first six PC axes of the bacterial community on each of the first four PC axis of the volatile profile for the same type of sample. PC factor were calculated after varimax normalized rotation. Each PC-axis was named by a composition of letters that indicate the type of samples. The first letter indicates whether the sample corresponds to bacteria (B) or volatiles (V), the second letter indicates whether the sample is from secretions of females (SF), nestlings (SN) or nest material (M). Finally, for types of samples that were collected at the beginning (1) and at the end (2) of the nestling period, the name finished with a number. **Table S7.** Results of PCA analyses summarizing bacterial genera, family and order, and volatiles detected in the nest material and the uropygial secretion of female and nestling hoopoes. Values are PC factor loadings after varimax normalized rotated and in their nest material or nest boxes. Each PC-axis was named by a composition of letters that indicate the type of samples. The first letter indicates whether the sample corresponds to bacteria (B) or volatiles (V), the second letter indicates whether the sample is from secretions of females (SF), nestlings (SN) or nest material (M). Finally, for types of samples that were collected at the beginning (1) and at the end (2) of the nestling period, the name finished with a number. Only factors than entered in final models explaining the association between bacterial and volatile profiles are shown. The six bacteria/volatile that better explained (positively and negatively) each axis are shown. **Table S8.** Multiple regression exploring the effect of the first four PC axes of the volatile profile of nestling and adult females on each of the first four PC axis of the volatile profile of the nest. PC factor were calculated after varimax normalized rotation. Each PC-axis was named by a composition of letters that indicate the type of samples. The first letter indicates corresponds to volatile samples (V), the second letter indicates whether the sample is from secretions of females (SF), nestlings (SN) or nest material (M). Finally, for types of samples that were collected at the beginning (1) and at the end (2) of the nestling period, the name finished with a number. **Table S9.** Results of PCA analyses summarizing volatiles detected in the nest material and the uropygial secretion of female and nestling hoopoes. Values are PC factor loadings after varimax normalized rotated and in their nest material or nest boxes. Each PC-axis was named by a composition of letters that indicate the type of samples. The first letter indicates whether the sample corresponds to bacteria (B) or volatiles (V), the second letter indicates whether the sample is from secretions of females (SF), nestlings (SN) or nest material (M). Finally, for types of samples that were collected at the beginning (1) and at the end (2) of the nestling period, the name finished with a number. Only factors than entered in final models explaining the association between volatile profiles of secretions and nest-box environment are shown. **Table S10.** Results of PCA analyses summarizing bacterial genera (if unknown we use family or order) and volatiles detected in the nest material and the uropygial secretion of female and nestling hoopoes that explained parasitism and fledging success. Values are PC factor loadings after varimax normalized rotated and in their nest material or nest boxes. Each PC-axis was named by a composition of letters that indicate the type of samples. The first letter indicates whether the sample corresponds to bacteria (B) or volatiles (V), the second letter indicates whether the sample is from secretions of females (SF), nestlings (SN) or nest material (M). Finally, for types of samples that were collected at the beginning (1) and at the end (2) of the nestling period, the name finished with a number. Only factors than entered in final models explaining the intensity of ecto-parasitism in females and nestlings and the fledging success are shown. N MAT refers to nest material and SECR to uropygial secretion.

**Additional file 2. Table S4. **Six first axes of Principal Component Analyses based on ASV table of bacterial community of different type of samples from hoopoes nests and secretions. **Table S5.** Six first axes of Principal Component Analyses based on volatile profile of different type of samples from hoopoes nests and secretions.

## Data Availability

Raw sequence reads and their metadata are deposited in the Sequence Read Archive (SRA) in the Genbank-NCBI webpage (https://www.ncbi.nlm.nih.gov/sra/ ). Nest sequences are available under BioProject ID: PRJNA847390, accession number, SUB11518282 and Secretion sequences under BioProject ID: PRJNA847428, accession number SUB11582000. Volatiles metadata are stored in DataDryad (10.5061/dryad.8sf7m0csn).
