# Supplementary material for: Volatiles of symbiotic bacterial origin explain ectoparasitism and fledging success of hoopoes
Source: Anim Microbiome. 2024 May 10;6:26. doi: 10.1186/s42523-024-00312-9 (PMC11084096; doi:10.1186/s42523-024-00312-9)
Supplement: Supplementary file 1 — Additional file 1. Table S1. Number of experimental (with autoclaved nest material), control (with non-autoclaved nest material) and natural (with old nest material) hoopoe nests with information of bacterial community, volatile profiles and parasitism at different nestling stages (day of sampling: days (d) after the first egg hatched). Bacterial and volatile samples were collected from the nest environment [nest material (Nest Mat) or nest air (Nest-box) and from the uropygial gland (secretion) of females and nestlings. Table S2. General Lineal Models exploring the effect of autoclaving nest material on Shannon (Sh) and Faith’s phylogenetic diversity (Pd) indexes of bacterial communities and volatile profiles at different nesting stages and nest locations. We show least square means (SE) for control and experimental (Exp) treatments, and for different study years, and beta (SE) values of the associations with laying date. Statistical effects lower than 0.05 are in bold. Table S3. General Lineal Models exploring the effect of using new nest boxes (i.e., Natural vs Control) on Shannon (Sh) and (Pd) alpha diversity indexes of bacterial communities and volatile profiles at different nesting stages and nest locations. We show least square means (SE) for control and experimental (Exp) treatments, and for different study years, and beta (SE) values of the associations with laying date. Statistical effects lower than 0.05 are in bold. Table S6. Multiple regression exploring the effect of the first six PC axes of the bacterial community on each of the first four PC axis of the volatile profile for the same type of sample. PC factor were calculated after varimax normalized rotation. Each PC-axis was named by a composition of letters that indicate the type of samples. The first letter indicates whether the sample corresponds to bacteria (B) or volatiles (V), the second letter indicates whether the sample is from secretions of females (SF), nestlings (SN) or nest material (M). [file 42523_2024_312_MOESM1_ESM.docx]

**Electronic Supplementary Material (ESM)**

**Table S1.** Number of experimental (with autoclaved nest material), control (with non-autoclaved nest material) and natural (with old nest material) hoopoe nests with information of bacterial community, volatile profiles and parasitism at different nestling stages (day of sampling: days (d) after the first egg hatched). Bacterial and volatile samples were collected from the nest environment [nest material (Nest Mat) or nest air (Nest-box) and from the uropygial gland (secretion) of females and nestlings.

|  | Visit | Experimental | Control | Natural |
| --- | --- | --- | --- | --- |
| **Bacterial community** |  |  |  |  |
| Nest material d14 | 3 | 34 | 33 | 22 |
| Nest material d15 | 4 | 30 | 25 | 14 |
| Female secretion d4 | 1 | 38 | 39 | 18 |
| Nestling secretion d19 | 7 | 34 | 30 | 12 |
| **Volatile profile** |  |  |  |  |
| Nest-box d7 | 5 | 30 | 29 | 3 |
| Nest-box d18 | 6 | 28 | 27 | 3 |
| Female secretion d4 | 1 | 43 | 41 | 13 |
| Nestling secretion d8 | 2 | 34 | 23 | 8 |
| Nestling secretion d19 |  | 43 | 36 | 11 |
| **Parasites** |  |  |  |  |
| Females d4 | 1 | 39 | 40 | 18 |
| Nestlings d18 | 6 | 36 | 25 | 6 |
| Nestlings d19 | 7 | 31 | 20 | 4 |

**Table S2.** General Lineal Models exploring the effect of autoclaving nest material on Shannon (Sh) and Faith’s phylogenetic diversity (Pd) indexes of bacterial communities and volatile profiles at different nesting stages and nest locations. We show least square means (SE) for control and experimental (Exp) treatments, and for different study years, and beta (SE) values of the associations with laying date. Statistical effects lower than 0.05 are in bold.

|  |  |  | Experimental treatment | | | |  | Study year | | | |  | Laying date | | |
| --- | --- | --- | --- | --- | --- | --- | --- | --- | --- | --- | --- | --- | --- | --- | --- |
|  |  |  | Exp  Mean (SE) | Control  Mean (SE) | F | P |  | 2017 | 2018 | F | P |  | Beta (SE) | F | P |
| **Bacterial communities** | | | | |  |  |  |  |  |  |  |  |  |  |  |
|  | *Nest material (day 4), d.f. = 1,63* | | | | | |  |  |  |  |  |  |  |  |  |
|  |  | Sh | **3.45 (0.21)** | **4.15 (0.21)** | **5.73** | **0.020** |  | 3.73 (0.23) | 3.87 (0.18) | 0.22 | 0.644 |  | -0.01 (0.13) | 0.01 | 0.978 |
|  |  | Pd | **10.35 (0.27)** | **11.96 (0.27)** | **18.0** | **0.0001** |  | 10.87 (0.30) | 11.44 (0.23) | 2.23 | 0.141 |  | -0.13 (0.12) | 1.26 | 0.266 |
|  | *Nest material (day 15), d.f. =1,50* | | | | | |  |  |  |  |  |  |  |  |  |
|  |  | Sh | 3.66 (0.18) | 4.07 (0.20) | 2.39 | 0.129 |  | 3.79 (0.22) | 3.94 (0.16) | 0.61 | 0.439 |  | 0.11 (0.15) | 0.61 | 0.439 |
|  |  | Pd | **9.36 (0.33)** | **10.49 (0.37)** | **5.19** | **0.027** |  | 9.80 (0.41) | 10.06 (0.29) | 0.27 | 0.606 |  | 0.10 (0.14) | 0.53 | 0.470 |
|  | *Female secretion (day 4), d.f. =1,74* | | | | | |  |  |  |  |  |  |  |  |  |
|  |  | Sh | 3.43 (0.05) | 3.42 (0.05) | 0.01 | 0.915 |  | 3.39 (0.05) | 3.47 (0.05) | 1.33 | 0.252 |  | 0.07 (0.12) | 0.37 | 0.543 |
|  |  | Pd | 6.18 (0.04) | 6.17 (0.04) | 0.02 | 0.896 |  | 6.19 (0.04) | 6.17 (0.05) | 0.08 | 0.780 |  | 0.18 (0.12) | 2.20 | 0.143 |
|  | *Nestling secretion (day 19), d.f. =1,61* | | | | | |  |  |  |  |  |  |  |  |  |
|  |  | Sh | 3.33 (0.05) | 3.29 (0.06) | 0.53 | 0.470 |  | 3.25 (0.05) | 3.37 (0.05) | 2.14 | 0.149 |  | -0.14 (0.14) | 0.01 | 0.907 |
|  |  | Pd | 6.41 (0.08) | 6.51 (0.09) | 0.20 | 0.658 |  | 6.37 (0.08) | 6.55 (0.08) | 2.90 | 0.094 |  | -0.02 (0.14) | 0.98 | 0.327 |
| **Volatile profiles** | | | |  |  |  |  |  |  |  |  |  |  |  |  |
|  | *Nest-boxes (day7), d.f. = 1,55* | | | | | |  |  |  |  |  |  |  |  |  |
|  |  | Sh | **2.37 (0.02)** | **2.29 (0.02)** | **5.43** | **0.023** |  | **2.22 (0.03)** | **2.44 (0.02)** | **43.3** | **0.001** |  | **-0.22 (0.10)** | **5.04** | **0.029** |
|  | *Nest-boxes (day18), d.f. = 1,51* | | | | | |  |  |  |  |  |  |  |  |  |
|  |  | Sh | 2.38 (0.03) | 2.32 (0.03) | 1.51 | 0.226 |  | **2.26 (0.04)** | **2.44 (0.03)** | **15.8** | **0.001** |  | -0.21 (0.13) | 2.84 | 0.098 |
|  | *Female secretion (day 4), d.f. = 1,80* | | | | | |  |  |  |  |  |  |  |  |  |
|  |  | Sh | 2.22 (0.05) | 2.24 (0.05) | 0.13 | 0.720 |  | 0.23 (0.05) | 0.23 (0.05) | 0.00 | 0.992 |  | -0.10 (0.12) | 0.65 | 0.424 |
|  | *Nestling secretion (day 8), d.f. = 1,63* | | | | | |  |  |  |  |  |  |  |  |  |
|  |  | Sh | 2.30 (0.04) | 2.32 (0.04) | 0.08 | 0.784 |  | 2.34 (0.04) | 2.28 (0.03) | 1.33 | 0.253 |  | 0.05 (0.136) | 0.12 | 0.734 |
|  | *Nestling secretion (day 19), d.f. = 1,76* | | | | | |  |  |  |  |  |  |  |  |  |
|  |  | Sh | 2.26 (0.04) | 2.29 (0.04) | 0.33 | 0.566 |  | 2.27 (0.04) | 2.28 (0.04) | 0.02 | 0.901 |  | -0.18 (0.12) | 2.16 | 0.145 |

**Table S3.** General Lineal Models exploring the effect of using new nest boxes (i.e., Natural vs Control) on Shannon (Sh) and (Pd) alpha diversity indexes of bacterial communities and volatile profiles at different nesting stages and nest locations. We show least square means (SE) for control and experimental (Exp) treatments, and for different study years, and beta (SE) values of the associations with laying date. Statistical effects lower than 0.05 are in bold.

|  |  |  | Experimental treatment | | | |  | Study year | | | |  | Laying date | | |
| --- | --- | --- | --- | --- | --- | --- | --- | --- | --- | --- | --- | --- | --- | --- | --- |
|  |  |  | Natural  Mean (SE) | Control  Mean (SE) | F | P |  | 2017 | 2018 | F | P |  | Beta (SE) | F | P |
| **Bacterial communities** | | | | |  |  |  |  |  |  |  |  |  |  |  |
|  | *Nest material (day 4), d.f. = 1,42* | | | | | |  |  |  |  |  |  |  |  |  |
|  |  | Sh | 3.64 (0.26) | 4.15 (0.19) | 2.92 | 0.095 |  | 3.94 (0.25) | 3.88 (0.19) | 0.03 | 0.863 |  | -0.03 (0.15) | 0.03 | 0.865 |
|  |  | Pd | **9.75 (0.30)** | **11.96 (0.22)** | **36.0** | **0.0001** |  | 10.74 (0.30) | 10.97 (0.23) | 0.38 | 0.541 |  | -0.09 (0.11) | 0.58 | 0.451 |
|  | *Nest material (day 15), d.f. =1,33* | | | | | |  |  |  |  |  |  |  |  |  |
|  |  | Sh | **3.37 (0.24)** | **4.21 (0.18)** | **7.49** | **0.010** |  | 3.90 (0.23) | 0.69 (0.23) | 0.54 | 0.470 |  | -0.21 (0.16) | 1.61 | 0.214 |
|  |  | Pd | **9.54 (0.39)** | **10.76 (0.29)** | **6.03** | **0.020** |  | 10.49 (9.81) | 9.81 (0.30) | 2.06 | 0.161 |  | -0.17 (0.16) | 1.08 | 0.307 |
|  | *Female secretion (day 4), d.f. =1,53* | | | | | |  |  |  |  |  |  |  |  |  |
|  |  | Sh | 3.44 (0.07) | 3.43 (0.05) | 0.04 | 0.843 |  | 3.42 (0.06) | 3.45 (0.05) | 0.12 | 0.731 |  | 0.12 (0.14) | 0.76 | 0.386 |
|  |  | Pd | 6.30 (0.06) | 6.18 (0.04) | 2.55 | 0.116 |  | 6.23 (0.05) | 6.25 (0.05) | 0.05 | 0.817 |  | 0.25 (0.13) | 3.71 | 0.060 |
|  | *Nestling secretion (day 19), d.f. =1,39* | | | | | |  |  |  |  |  |  |  |  |  |
|  |  | Sh | 3.29 (0.09) | 3.28 (0.06) | 0.01 | 0.964 |  | 3.25 (0.08) | 3.31 (0.06) | 0.34 | 0.561 |  | -0.15 (0.16) | 0.85 | 0.363 |
|  |  | Pd | 6.47 (0.10) | 6.51 (0.06) | 0.10 | 0.759 |  | 6.52 (0.09) | 6.46 (0.07) | 0.39 | 0.535 |  | -0.08 (0.16) | 0.22 | 0.640 |
| **Volatile profiles** | | | |  |  |  |  |  |  |  |  |  |  |  |  |
|  | *Nest-boxes (day7), d.f. = 1,28* | | | | | |  |  |  |  |  |  |  |  |  |
|  |  | Sh | 2.39 (0.08) | 2.29 (0.02) | 1.51 | 0.230 |  | **2.23 (0.04)** | **2.45 (0.04)** | **24.4** | **0.0001** |  | **-0.38 (0.14)** | **7.24** | **0.012** |
|  | *Nest-boxes (day18), d.f. = 1,26* | | | | | |  |  |  |  |  |  |  |  |  |
|  |  | Sh | 2.38 (0.10) | 2.32 (0.03) | 0.27 | 0.607 |  | **2.28 (0.06)** | **2.42 (0.06)** | **5.23** | **0.031** |  | -0.29 (0.19) | 2.36 | 0.137 |
|  | *Female secretion (day 4), d.f. = 1,50* | | | | | |  |  |  |  |  |  |  |  |  |
|  |  | Sh | 2.06 (0.11I | 2.23 (0.05) | 2.02 | 0.162 |  | 2.11 (0.10) | 2.18 (0.06) | 0.36 | 0.552 |  | -0.05 (0.15) | 0.13 | 0.717 |
|  | *Nestling secretion (day 8), d.f. = 1,37* | | | | | |  |  |  |  |  |  |  |  |  |
|  |  | Sh | 2.33 (0.08) | 2.32 (0.03) | 0.01 | 0.972 |  | **2.40 (0.07)** | **2.25 (0.04)** | **4.74** | **0.036** |  | 0.05 (0.16) | 0.11 | 0.740 |
|  | *Nestling secretion (day 19), d.f. = 1,43* | | | | | |  |  |  |  |  |  |  |  |  |
|  |  | Sh | 2.32 (0.09) | 2.29 (0.05) | 0.10 | 0.757 |  | 2.30 (0.08) | 2.31 (0.06 | 0.01 | 0.929 |  | -0.16 (0.16) | 0.98 | 0.328 |

**Table S6.** Multiple regression exploring the effect of the first six PC axes of the bacterial community on each of the first four PC axis of the volatile profile for the same type of sample. PC factor were calculated after varimax normalized rotation. Each PC-axis was named by a composition of letters that indicate the type of samples. The first letter indicates whether the sample corresponds to bacteria (B) or volatiles (V), the second letter indicates whether the sample is from secretions of females (SF), nestlings (SN) or nest material (M). Finally, for types of samples that were collected at the beginning (1) and at the end (2) of the nestling period, the name finished with a number.

|  | *d.f.* | *F* | *Padj* | *F* | *Padj* | *F* | *Padj* | *F* | *Padj* | *F* | *Padj* | *F* | *Padj* |
| --- | --- | --- | --- | --- | --- | --- | --- | --- | --- | --- | --- | --- | --- |
| Female secretion (SF) | | **PC1-BSF** | | **PC2-BSF** | | **PC3-BSF** | | **PC4-BSF** | | **PC5-BSF** | | **PC6-BSF** | |
| **PC1-VSF** | 1,63 | 0.06 | 0.806 | 2.25 | 0.138 | 0.07 | 0.789 | 0.35 | 0.557 | **9.02** | **0.015** | 0.04 | 0.839 |
| **PC2-VSF** | 1,63 | 0.67 | 0.416 | **5.75** | **0.039** | 0.79 | 0.376 | 0.94 | 0.337 | 2.30 | 0.134 | 0.09 | 0.765 |
| **PC3-VSF** | 1,63 | 1.04 | 0.311 | 0.01 | 0.934 | 0.06 | 0.808 | 1.46 | 0.232 | 0.04 | 0.839 | 0.46 | 0.502 |
| **PC4-VSF** | 1,63 | 0.11 | 0.738 | 3.42 | 0.069 | **6.19** | **0.016** | 1.26 | 0.265 | 2.62 | 0.111 | 2.08 | 0.155 |
| Nesting secretion (SN) | | **PC1-BSN2** | | **PC2-BSN2** | | **PC3-BSN2** | | **PC4-BSN2** | | **PC5-BSN2** | | **PC6-BSN2** | |
| **PC1-VSN2** | 1,58 | 2.18 | 0.145 | 0.28 | 0.599 | 0.02 | 0.884 | 2.20 | 0.143 | 1.16 | 0.286 | 0.47 | 0.495 |
| **PC2-VSN2** | 1,58 | 1.85 | 0.179 | 0.01 | 0.912 | **6.46** | **0.018** | 0.44 | 0.510 | 0.29 | 0.594 | 0.58 | 0.448 |
| **PC3-VSN2** | 1,58 | 0.13 | 0.715 | 1.70 | 0.197 | **10.73** | **0.004** | **10.79** | **0.007** | 0.01 | 0.917 | 0.41 | 0.525 |
| **PC4-VSN2** | 1,58 | 0.18 | 0.672 | 0.13 | 0.719 | 0.49 | 0.487 | 0.49 | 0.487 | 0.25 | 0.618 | 0.63 | 0.430 |
| Nest material (N1) | | **PC1-BM1** | | **PC2-BM1** | | **PC3-BM1** | | **PC4-BM1** | | **PC5-BM1** | | **PC6-BM1** | |
| **PC1-VM1** | 1,34 | 0.25 | 0.620 | 0.12 | 0.727 | 0.81 | 0.376 | 0.00 | 0.971 | 0.01 | 0.907 | **8.57** | **0.024** |
| **PC2-VM1** | 1,34 | 0.02 | 0.888 | 0.91 | 0.348 | 1.14 | 0.294 | 0.02 | 0.884 | 0.02 | 0.884 | 2.77 | 0.105 |
| **PC3-VM1** | 1,34 | 0.08 | 0.778 | 0.58 | 0.451 | 0.45 | 0.509 | 0.12 | 0.731 | 3.04 | 0.090 | 0.75 | 0.394 |
| **PC4-VM1** | 1,34 | 0.00 | 0.996 | 0.66 | 0.422 | 0.97 | 0.331 | 0.00 | 0.955 | 2.30 | 0.138 | 0.40 | 0.532 |
| Nest material (N2) | | **PC1-BM2** | | **PC2-BM2** | | **PC3-BM2** | | **PC4-BM2** | | **PC5-BM2** | | **PC6-BM2** | |
| **PC1-VM2** | 1,32 | 0.80 | 0.376 | 1.34 | 0.256 | 3.04 | 0.091 | 0.57 | 0.455 | 0.01 | 0.913 | 0.05 | 0.828 |
| **PC2-VM2** | 1,32 | 0.00 | 0.951 | **4.91** | **0.045** | 0.31 | 0.580 | 0.16 | 0.687 | 0.06 | 0.802 | 0.91 | 0.348 |
| **PC3-VM2** | 1,32 | 0.30 | 0.586 | 3.32 | 0.078 | 1.06 | 0.310 | 0.81 | 0.376 | 3.22 | 0.082 | 5.60 | 0.097 |
| **PC4-VM2** | 1,32 | 0.80 | 0.378 | 5.18 | 0.059 | 0.03 | 0.866 | 0.05 | 0.820 | **4.44** | **0.043** | 0.14 | 0.713 |

**Table S7**. Results of PCA analyses summarizing bacterial genera, family and order, and volatiles detected in the nest material and the uropygial secretion of female and nestling hoopoes. Values are PC factor loadings after varimax normalized rotated and in their nest material or nest boxes. Each PC-axis was named by a composition of letters that indicate the type of samples. The first letter indicates whether the sample corresponds to bacteria (B) or volatiles (V), the second letter indicates whether the sample is from secretions of females (SF), nestlings (SN) or nest material (M). Finally, for types of samples that were collected at the beginning (1) and at the end (2) of the nestling period, the name finished with a number. Only factors than entered in final models explaining the association between bacterial and volatile profiles are shown. The six bacteria/volatile that better explained (positively and negatively) each axis are shown.

| FEMALE UROPYGIAL SECRETION (MODEL: VOLATILES ~ BACTERIA) | | | | | | | | | |
| --- | --- | --- | --- | --- | --- | --- | --- | --- | --- |
|  |  | **PC2** | **PC3** | **PC5** |  | **PC1** | **PC2** | **PC4** |  |
| **Phylum** | **Bacterial Orden-Family-Genus** | **BSF** | **BSF** | **BSF** |  | **VSF** | **VSF** | **VSF** | **Volatile Components** |
| Firmicutes | Clostridiales – Ruminococcaceae – unknown genus | -0.48 |  |  |  | -0.84 |  | 0.29 | Phenol |
| Firmicutes | Clostridiales – Tissierellaceae – GW-34 | -0.32 |  |  |  | -0.31 |  |  | Dtrisulfide |
| Firmicutes | Clostridiales – Veillonellaceae – *Dialister* strain 1 | -0.31 |  |  |  | -0.21 |  | 0.57 | 4 methyl pentanoic acid methyl ester |
| Firmicutes | Erysipelotrichales – Erysipelotrichaceae – unknown genus 5 | 0.63 |  |  |  | 0.93 |  |  | Hexanal |
| Proteobacteria | Pasteurellales – Pasteurellaceae – Haemophilus | 0.68 |  |  |  | 0.93 |  |  | Heptanal |
| Firmicutes | Clostridiales – Peptostreptococcaceae – unknown genus | 0.74 |  |  |  | 0.95 |  |  | Octanal |
| Firmicutes | Clostridiales – Tissierellaceae – *Helcococcus* |  | -0.66 |  |  |  | -0.79 |  | Nonoic acid methyl ester |
| Firmicutes | Clostridiales – Clostridiaceae – *Clostridium* |  | -0.46 |  |  |  | -0.73 |  | Butanoic acid methyl ester |
| Firmicutes | Erysipelotrichales – Erysipelotrichaceae – unknown genus 2 |  | -0.43 |  |  |  | -0.70 |  | Acetic acid |
| Firmicutes | Clostridiales – Tissierellaceae – *Parvimonas* |  | 0.39 |  |  |  | 0.66 |  | 3 methyl butanoic acid ethyl ester |
| Firmicutes | Erysipelotrichales – Erysipelotrichaceae – unknown genus 4 |  | 0.41 |  |  |  | 0.66 |  | Hexanoic acid ethyl ester |
| Firmicutes | Clostridiales – Tissierellaceae – *Helcococcus* |  | 0.61 |  |  |  | 0.77 |  | Butanoic acid ethyl ester |
| Firmicutes | Clostridiales – Mogibacteriaceae – unknown genus 3 |  |  | -0.49 |  |  |  | -0.63 | Isovaleric acid |
| Firmicutes | Clostridiales Tissierellaceae – unknown genus 2 |  |  | -0.44 |  |  |  | -0.61 | Butanoic acid |
| Firmicutes | Erysipelotrichales – Erysipelotrichaceae – unknown genus 1 |  |  | -0.42 |  |  |  | -0.60 | Octanoic acid |
| Firmicutes | Clostridiales – Veillonellaceae – *Dialister* strain 2 |  |  | 0.48 |  |  |  | 0.29 | Benzaldehide |
| Firmicutes | Clostridiales – Tissierellaceae – unknown genus 5 |  |  | 0.51 |  |  |  |  |  |
| Firmicutes | Clostridiales – Tissierellaceae – *Anaerococcus* |  |  | 0.60 |  |  |  |  |  |
|  | **Explained Variance (%)** | **5.92** | **6.14** | **5.39** |  | **25.30** | **18.27** | **9.87** |  |
|  |  |  |  |  |  |  |  |  |  |
| NESTLING UROPYGIAL SECRETION at late stage of nestlings (VOLATILES ~ BACTERIA) | | | | | | | | |  |
|  |  | **PC3** | **PC4** |  |  | **PC2** | **PC3** |  |  |
| **Phylum** | **Bacterial Orden-Family-Genus** | **BSN2** | **BSN2** |  |  | **VSN2** | **VSN2** |  | **Volatile Components** |
| Firmicutes | Erysipelotrichales – Erysipelotrichaceae – unknown genus 2 | -0.47 |  |  |  | -0.90 |  |  | Butanoic acid ethyl ester |
| Firmicutes | Clostridiales – Veillonellaceae – *Dialister* | -0.47 |  |  |  | -0.84 |  |  | 3 methyl butanoic acid ethyl ester |
| Bacteroidetes | Bacteroidales – Porphyromonadaceae – *Porphyromonas* | -0.45 |  |  |  | -0.82 |  |  | Hexanoic acid ethyl ester |
| Firmicutes | Clostridiales – Ruminococcaceae – unknown genus | 0.49 |  |  |  | 0.59 | 0.49 |  | Nonanoic acid methyl ester |
| Proteobacteria | Clostridiales – unknown family – unknown genus 2 | 0.55 |  |  |  | 0.68 |  |  | 4 methyl pentanoic acid methyl ester |
| Actinobacteria | Coriobacteriales – Coriobacteriaceae – unknown genus | 0.69 |  |  |  | 0.68 | 0.40 |  | Heptanoic acid methyl ester |
| Firmicutes | Erysipelotrichales – Erysipelotrichaceae – unknown genus 3 |  | -0.65 |  |  |  | -0.90 |  | Heptanoic acid |
| Firmicutes | Clostridiales – Tissierellaceae- *Peptoniphilus* |  | -0.43 |  |  |  | -0.88 |  | Hexanoic acid |
| Firmicutes | Clostridiales – Peptostreptococcaceae – unknown genus 3 |  | -0.34 |  |  |  | -0.75 |  | Pentanoic acid |
| Firmicutes | Clostridiales – Tissierellaceae – GW-34 strain 3 |  | 0.47 |  |  |  | 0.52 |  | Butanoic acid methyl ester |
| Actinobacteria | Solirubrobacterales – Conexibacteraceae – unknown genus |  | 0.58 |  |  |  |  |  |  |
| Firmicutes | Clostridiales – Tissierellaceae – *Parvimonas* |  | 0.65 |  |  |  |  |  |  |
|  | **Explained Variance (%)** | **6.83** | **5.96** |  |  | **17.46** | **14.32** |  |  |
|  |  |  |  |  |  |  |  |  |  |
| HOOPOE NESTS at early stage of nestlings (MODEL: VOLATILES ~ BACTERIA) | | | | | | |  |  |  |
|  |  | **PC1** |  |  |  | **PC6** |  |  |  |
| **Phylum** | **Bacterial Orden-Family-Genus** | **BM1** |  |  |  | **VM1** |  |  | **Volatile components** |
| Proteobacteria | Pasteurellales – Pasteurellaceae – *Haemophilus* | -0.29 |  |  |  | -0.90 |  |  | Hexanoic acid |
| Firmicutes | Lactobacillales -Streptococcaceae – *Lactococcus* | -0.24 |  |  |  | -0.54 |  |  | Pentanoic acid |
| Proteobacteria | Aeromonadales – Aeromonadaceae – unknwon genus | -0.24 |  |  |  | -0.41 |  |  | Propionic acid |
| Actinobacteria | Actinomycetales – Corynebacteriaceae – *Corynebacterium* | 0.63 |  |  |  | 0.21 |  |  | Isocaproic acid |
| Proteobacteria | Rhodobacterales – Rhodobacteraceae – *Paracoccus* | 0.65 |  |  |  | 0.38 |  |  | Butanoic acid |
| Actinobacteria | Actinomycetales – Micrococcaceae – *Rothia* | 0.66 |  |  |  | 0.68 |  |  | Benzaldehide |
|  | **Explained Variance (%)** | **7.36** |  |  |  | **16.97** |  |  |  |
|  |  |  |  |  |  |  |  |  |  |
| HOOPOE NESTS at late stage of nestlings (MODEL: VOLATILES ~ BACTERIA) | | | | | | |  |  |  |
|  |  | **PC2** | **PC5** |  |  | **PC2** | **PC4** |  |  |
| **Phylum** | **Bacterial Orden-Family-Genus** | **BM2** | **BM2** |  |  | **VM2** | **VM2** |  | **Volatile components** |
| Bacteroidetes | Bacteroidales – Porphyromonadaceae – *Porphyromonas* | -0.69 |  |  |  | -0.73 |  |  | Butanoic acid |
| Proteobacteria | Rhizobiales -Brucellaceae – *Ochrobactrum* | -0.66 |  |  |  | -0.53 | -0.22 |  | Acetic acid |
| Fusobacteria | Fusobacteriales – Leptotrichiaceae – unknown genus | -0.65 |  |  |  | -0.26 |  |  | Benzaldehide |
| Firmicutes | Bacillales – Planococcaceae – *Sporosarcina* strain 7 | 0.33 |  |  |  | 0.68 |  |  | Octanal |
| Firmicutes | Bacillales – Planococcaceae – Sporosarcina strain 6 | 0.36 |  |  |  | 0.75 |  |  | Pentanal |
| Firmicutes | Bacillales – Staphylococcaceae – *Jeotgalicoccus* | 0.40 |  |  |  | 0.88 |  |  | Hexanal |
| Bacteroidetes | Cytophagales – Flammeovirgaceae – *Roseivirga* |  | -0.42 |  |  |  | -0.37 |  | Hexanoic acid |
| Firmicutes | Clostridiales – Tissierellaceae – *Peptoniphilus* |  | -0.37 |  |  |  | -0.35 |  | Decanoic acid |
| Bacteroidetes | Sphingobacteriales – Sphingobacteriaceae – unknown genus 2 |  | -0.37 |  |  |  | 0.70 |  | Isocaproic acid |
| Firmicutes | Clostridiales – Tissierellaceae – *Helcococcus* |  | 0.69 |  |  |  | 0.75 |  | Isobutiric acid |
| Firmicutes | Bacillales – Planococcaceae – *Sporosarcina* strain 5 |  | 0.71 |  |  |  | 0.79 |  | Isovaleric acid |
| Firmicutes | Bacillales – Planococcaceae – *Sporosarcina* strain 3 |  | 0.72 |  |  |  |  |  |  |
|  | **Explained Variance (%)** | **6.39** | **5.97** |  |  | **20.97** | **11.89** |  |  |

**Table S8.** Multiple regression exploring the effect of the first four PC axes of the volatile profile of nestling and adult females on each of the first four PC axis of the volatile profile of the nest. PC factor were calculated after varimax normalized rotation. Each PC-axis was named by a composition of letters that indicate the type of samples. The first letter indicates corresponds to volatile samples (V), the second letter indicates whether the sample is from secretions of females (SF), nestlings (SN) or nest material (M). Finally, for types of samples that were collected at the beginning (1) and at the end (2) of the nestling period, the name finished with a number.

|  |  | *d.f.* | *F* | *Padj* | *F* | *Padj* | *F* | *Padj* | *F* | *Padj* | |
| --- | --- | --- | --- | --- | --- | --- | --- | --- | --- | --- | --- |
|  |  |  | **PC1-VSF** | | **PC2-VSF** | | **PC3-VSF** | | **PC4-VSF** | |  |
|  | **PC1-VM1** | **1,50** | **4.37** | **0.042** | **10.01** | **0.007** | 0.40 | 0.529 | 0.00 | 0.996 | |
|  | **PC2-VM1** | 1,50 | 0.03 | 0.860 | 0.00 | 0.962 | 0.10 | 0.751 | 0.01 | 0.930 | |
|  | **PC3-VM1** | 1,50 | 0.23 | 0.634 | 1.26 | 0.266 | 0.30 | 0.584 | 0.57 | 0.453 | |
|  | **PC4-VM1** | **1,50** | **6.48** | **0.019** | **9.48** | **0.007** | **11.19** | **0.013** | 0.14 | 0.705 | |
|  |  |  | **PC1-VSN1** | | **PC2-VSN1** | | **PC3-VSN1** | | **PC4-VSN1** | |  |
|  | **PC1-VM1** | **1,43** | 1.24 | 0.271 | **4.49** | **0.046** | **11.13** | **0.007** | 0.10 | 0.749 | |
|  | **PC2-VM1** | 1,43 | 0.05 | 0.824 | 0.25 | 0.617 | **4.30** | **0.044** | 0.09 | 0.762 | |
|  | **PC3-VM1** | 1,43 | 0.55 | 0.460 | 3.31 | 0.076 | 1.30 | 0.261 | 0.53 | 0.471 | |
|  | **PC4-VM1** | **1,43** | 0.01 | 0.908 | 0.54 | 0.465 | **8.95** | **0.007** | 3.06 | 0.087 | |
|  |  |  |  |  |  |  |  |  |  |  |  |
|  |  |  | **PC1-VSF** | | **PC2-VSF** | | **PC3-VSF** | | **PC4-VSF** | | |
|  | **PC1-VM2** | **1,47** | 1.10 | 0.299 | **6.62** | **0.021** | **4.64** | **0.036** | 0.04 | 0.836 | |
|  | **PC2-VM2** | **1,47** | **9.29** | **0.010** | **27.35** | **0.000** | **7.71** | **0.016** | 1.38 | 0.246 | |
|  | **PC3-VM2** | 1,47 | 0.00 | 0.975 | 1.91 | 0.173 | 0.26 | 0.613 | 0.05 | 0.831 | |
|  | **PC4-VM2** | 1,47 | 0.49 | 0.486 | 0.49 | 0.486 | 1.88 | 0.177 | 0.07 | 0.790 | |
|  |  |  | **PC1-VSN2** | | **PC2-VSN2** | | **PC3-VSN2** | | **PC4-VSN2** | |  |
|  | **PC1-VM2** | 1,47 | 1.77 | 0.190 | 1.37 | 0.248 | 1.51 | 0.225 | 2.84 | 0.099 | |
|  | **PC2-VM2** | **1,47** | **5.20** | **0.031** | **46.36** | **0.000** | 0.31 | 0.578 | 1.18 | 0.283 | |
|  | **PC3-VM2** | 1,47 | 2.88 | 0.096 | 2.03 | 0.161 | 0.11 | 0.741 | 0.07 | 0.790 | |
|  | **PC4-VM2** | **1,47** | 1.60 | 0.211 | 0.75 | 0.390 | **6.59** | **0.018** | 0.47 | 0.495 | |

**Table S9**. Results of PCA analyses summarizing volatiles detected in the nest material and the uropygial secretion of female and nestling hoopoes. Values are PC factor loadings after varimax normalized rotated and in their nest material or nest boxes. Each PC-axis was named by a composition of letters that indicate the type of samples. The first letter indicates whether the sample corresponds to bacteria (B) or volatiles (V), the second letter indicates whether the sample is from secretions of females (SF), nestlings (SN) or nest material (M). Finally, for types of samples that were collected at the beginning (1) and at the end (2) of the nestling period, the name finished with a number. Only factors than entered in final models explaining the association between volatile profiles of secretions and nest-box environment are shown.

| AT THE BEGINNING OF THE NESTING PERIOD | | | | | | | | | | | | | | | | | | | | | | |
| --- | --- | --- | --- | --- | --- | --- | --- | --- | --- | --- | --- | --- | --- | --- | --- | --- | --- | --- | --- | --- | --- | --- |
|  |  | FEMALES | | | | | | | | | |  | NESTLINGS | | | | | | | | | |
|  |  | SECRETION | | | | |  | | NESTS | | |  | SECRETION | | | |  | NESTS | | | | |
|  |  | **PC1** | **PC2** | **PC3** | | | |  | **PC1** | | **PC4** |  | **PC2** | | **PC3** |  |  | **PC1** | **PC2** | | | **PC4** |
|  | **Volatile profile** | **VSF** | **VSF** | **VSF** | | | |  | **VM1** | | **VM1** |  | **VSN1** | | **VSN1** |  |  | **VM1** | **VM1** | | **VM1** | |
| ***Acids*** | Acetic |  | -0.70 |  | | | |  |  | -0.39 | |  |  |  | |  |  |  | -0.39 |  | | |
| ***Acids*** | Butanoic |  |  |  | | | |  | 0.38 | -0.75 | |  |  |  | |  |  | 0.38 | -0.75 |  | | |
| ***Acids*** | Heptanoic |  |  |  | | | |  |  |  | |  |  | -0.90 | |  |  |  |  |  | | |
| ***Acids*** | Hexanoic |  |  |  | | | |  | -0.90 |  | |  |  | -0.88 | |  |  | -0.90 |  |  | | |
| ***Acids*** | Isobutiric |  |  | 0.91 | | | |  |  | 0.23 | |  |  |  | |  |  |  | 0.23 |  | | |
| ***Acids*** | Isocaproic |  |  | 0.74 | | | |  | 0.21 |  | |  |  |  | |  |  | 0.21 |  | -0.61 | | |
| ***Acids*** | Isovaleric |  |  |  | | | |  |  |  | |  |  |  | |  |  |  |  | 0.64 | | |
| ***Acids*** | Pentanoic |  |  | 0.81 | | | |  | -0.54 |  | |  |  | -0.75 | |  |  | -0.54 |  |  | | |
| ***Acids*** | Propianoic |  |  |  | | | |  | -0.41 |  | |  |  |  | |  |  | -0.41 |  |  | | |
| ***Sulfides*** | Ddisulfide |  |  | -0.18 | | | |  |  |  | |  |  |  | |  |  |  |  |  | | |
| ***Sulfides*** | Dtrisulfide | -0.31 |  | -0.27 | | | |  |  |  | |  |  |  | |  |  |  |  | -0.50 | | |
| ***Aldehydes*** | Benzaldehyde |  |  | -0.21 | | | |  | 0.68 | -0.24 | |  |  |  | |  |  | 0.68 | -0.24 | -0.42 | | |
| ***Aldehydes*** | Heptanal | 0.93 |  |  | | | |  |  | 0.48 | |  |  |  | |  |  |  | 0.48 |  | | |
| ***Aldehydes*** | Hexanal | 0.93 |  |  | | | |  |  | 0.81 | |  |  |  | |  |  |  | 0.81 |  | | |
| ***Aldehydes*** | Octanal | 0.95 |  |  | | | |  |  |  | |  |  |  | |  |  |  |  |  | | |
| ***Esters*** | 3-mehyl-butanoic-acid-ethyl-ester |  | 0.66 |  | | | |  |  |  | |  | -0.84 |  | |  |  |  |  |  | | |
| ***Esters*** | 4methyl-pentanoic-acid-methyl-ester | -0.21 |  |  | | | |  |  |  | |  | 0.68 |  | |  |  |  |  |  | | |
| ***Esters*** | Butanoic-acid-ethyl-ester |  | 0.77 |  | | | |  |  |  | |  | -0.90 |  | |  |  |  |  |  | | |
| ***Esters*** | Butanoic-acid-methyl-ester |  | -0.73 |  | | | |  |  |  | |  |  | 0.52 | |  |  |  |  |  | | |
| ***Esters*** | Hexanoic-acid-ethy-lester |  | 0.66 |  | | | |  |  |  | |  | -0.82 |  | |  |  |  |  |  | | |
| ***Esters*** | Heptanoic-acid-methyl-ester |  |  |  | | | |  |  |  | |  | 0.68 | 0.40 | |  |  |  |  |  | | |
| ***Esters*** | Nonoicacidmethylester |  | -0.79 |  | | | |  |  |  | |  | 0.59 | 0.49 | |  |  |  |  | 0.40 | | |
| ***Aromatic organic***  ***compounds*** | Phenol | -0.84 |  |  | | | |  |  |  | |  |  |  | |  |  |  |  | 0.48 | | |
|  | **Explained Variance (%)** | **25.30** | **18.27** | | | **12.27** | |  | **16.97** | **12.50** | |  | **14.32** | **8.94** | |  |  | **16.97** | **22.25** | **23.71** | | |
|  |  |  |  | | |  | |  |  |  | |  |  |  | |  |  |  |  |  | | |
| AT THE END OF THE NESTING PERIOD | | | | | | | | | | | | | | | | | | | | | | |
|  |  | FEMALES | | | | | | | | | |  | NESTLINGS | | | | | | | | | |
|  |  | SECRETION | | | | | |  | NESTS | | |  | SECRETION | | | |  | NESTS | | | | |
|  |  | **PC1** | **PC2** | | **PC3** | | |  | **PC1** | **PC2** | |  | **PC1** | **PC2** | | **PC3** |  | **PC2** | **PC4** |  | | |
|  | **Volatile profile** | **VSF** | **VSF** | | **VSF** | | |  | **VM2** | **VM2** | |  | **VSN2** | **VSN2** | | **VSN2** |  | **VM2** | **VM2** |  | | |
| ***Acids*** | Acetic |  | -0.70 | |  | | |  |  | -0.53 | |  |  |  | |  |  | -0.53 | -0.22 |  | | |
| ***Acids*** | Butanoic |  |  | |  | | |  |  | -0.73 | |  |  |  | |  |  | -0.73 |  |  | | |
| ***Acids*** | Decanoic |  |  | |  | | |  | 0.69 |  | |  |  |  | |  |  |  | -0.35 |  | | |
| ***Acids*** | Heptanoic |  |  | |  | | |  |  |  | |  |  | 0.59 | |  |  |  |  |  | | |
| ***Acids*** | Hexanoic |  |  | |  | | |  | -0.39 |  | |  |  |  | |  |  |  | -0.37 |  | | |
| ***Acids*** | Isobutiric |  |  | | 0.91 | | |  |  |  | |  |  |  | |  |  |  | 0.75 |  | | |
| ***Acids*** | Isocaproic |  |  | | 0.74 | | |  |  |  | |  |  |  | |  |  |  | 0.70 |  | | |
| ***Acids*** | Isovaleric |  |  | |  | | |  |  |  | |  |  |  | |  |  |  | 0.79 |  | | |
| ***Acids*** | Nonanoic |  |  | |  | | |  | 0.83 |  | |  |  |  | | -0.51 |  |  |  |  | | |
| ***Acids*** | Octanoic |  |  | |  | | |  |  |  | |  |  |  | | -0.52 |  |  |  |  | | |
| ***Acids*** | Pentanoic |  |  | | 0.81 | | |  | -0.73 |  | |  |  |  | |  |  |  |  |  | | |
| ***Acids*** | Propianoic |  |  | |  | | |  | -0.75 |  | |  | -0.26 |  | | -0.56 |  |  |  |  | | |
| ***Sulfides*** | Ddisulfide |  |  | | -0.18 | | |  |  |  | |  |  |  | | 0.67 |  |  |  |  | | |
| ***Sulfides*** | Dtrisulfide | -0.31 |  | | -0.27 | | |  |  |  | |  |  |  | | 0.69 |  |  |  |  | | |
| ***Aldehydes*** | Benzaldehyde |  |  | | -0.21 | | |  |  | -0.26 | |  |  |  | | 0.58 |  | -0.26 |  |  | | |
| ***Aldehydes*** | Heptanal | 0.93 |  | |  | | |  |  |  | |  | 0.93 |  | |  |  |  |  |  | | |
| ***Aldehydes*** | Hexanal | 0.93 |  | |  | | |  |  | 0.88 | |  | 0.92 |  | |  |  | 0.88 |  |  | | |
| ***Aldehydes*** | Octanal | 0.95 |  | |  | | |  |  | 0.68 | |  | 0.94 |  | |  |  | 0.68 |  |  | | |
| ***Aldehydes*** | Pentanal |  |  | |  | | |  |  | 0.75 | |  |  |  | |  |  | 0.75 |  |  | | |
| ***Esters*** | 3-mehyl-butanoic-acid-ethyl-ester |  | 0.66 | |  | | |  |  |  | |  |  |  | |  |  |  |  |  | | |
| ***Esters*** | 4-methyl-pentanoic-acid-methyl-ester | -0.21 |  | |  | | |  |  |  | |  |  |  | |  |  |  |  |  | | |
| ***Esters*** | Butanoic-acid-ethyl-ester |  | 0.77 | |  | | |  |  |  | |  |  | 0.72 | |  |  |  |  |  | | |
| ***Esters*** | Butanoic-acid-methyl-ester |  | -0.73 | |  | | |  |  |  | |  | -0.25 | -0.86 | |  |  |  |  |  | | |
| ***Esters*** | Heptanoic-acid-methyl-ester |  |  | |  | | |  |  |  | |  |  | -0.88 | |  |  |  |  |  | | |
| ***Esters*** | Hexanoic-acid-ethyl-ester |  | 0.66 | |  | | |  |  |  | |  |  | 0.62 | |  |  |  |  |  | | |
| ***Esters*** | Nonoic-acid-methyl-ester |  | -0.79 | |  | | |  |  |  | |  |  | -0.81 | |  |  |  |  |  | | |
| ***Aromatic organic***  ***compounds*** | Phenol | -0.84 |  | |  | | |  |  |  | |  | -0.86 |  | |  |  |  |  |  | | |
|  | **Explained Variance (%)** | **25.30** | **18.27** | | **12.27** | | |  | **16.32** | **20.97** | |  | **22.87** | **21.50** | | **12.44** |  | **20.97** | **11.89** |  | | |

**Table S10**. Results of PCA analyses summarizing bacterial genera (if unknown we use family or order) and volatiles detected in the nest material and the uropygial secretion of female and nestling hoopoes that explained parasitism and fledging success. Values are PC factor loadings after varimax normalized rotated and in their nest material or nest boxes. Each PC-axis was named by a composition of letters that indicate the type of samples. The first letter indicates whether the sample corresponds to bacteria (B) or volatiles (V), the second letter indicates whether the sample is from secretions of females (SF), nestlings (SN) or nest material (M). Finally, for types of samples that were collected at the beginning (1) and at the end (2) of the nestling period, the name finished with a number. Only factors than entered in final models explaining the intensity of ecto-parasitism in females and nestlings and the fledging success are shown. N MAT refers to nest material and SECR to uropygial secretion.

| PARASITISM OF 8 DAYS OLD NESTLINGS | | | | | | | | | | |
| --- | --- | --- | --- | --- | --- | --- | --- | --- | --- | --- |
|  |  | **NEST** | |  |  |  |  |  |  |  |
|  |  | **PC6** |  |  |  |  |  |  |  |  |
| **Phylum** | **Bacterial Orden – Family – Genus** | **BM1** |  |  |  |  |  |  |  |  |
|  | Pasteurellales – Pasteurellaceae – *Haemophilus* | -0.29 |  |  |  |  |  |  |  |  |
|  | Lactobacillales – Streptococcaceae – *Lactococcus* | -0.24 |  |  |  |  |  |  |  |  |
|  | Aeromonadales – Aeromonadaceae – unknown genus | -0.24 |  |  |  |  |  |  |  |  |
|  | Actinomycetales – Corynebacteriaceae – *Corynebacterium* | 0.63 |  |  |  |  |  |  |  |  |
|  | Rhodobacterales – Rhodobacteraceae – *Paracoccus* | 0.65 |  |  |  |  |  |  |  |  |
|  | Actinomycetales – Micrococcaceae – *Rothia* | 0.66 |  |  |  |  |  |  |  |  |
|  | **Explained Variance (%)** | **4.81** |  |  |  |  |  |  |  |  |
|  |  |  |  |  |  |  |  |  |  |  |
| PARASITISM OF 19 DAYS OLD NESTLINGS | | | | | | | | | | |
|  |  | **NESTLINGS** | |  |  |  |  | |  |  |
|  |  | **SECRETIONS** | |  |  |  | **NEST** | |  |  |
|  |  | **PC2** | **PC4** |  |  |  | **PC2** | **PC3** |  |  |
| **Phylum** | **Bacterial Orden – Family – Genus** | **BSN2** | **BSN2** |  |  |  | **VM2** | **VM2** |  | **Volatile profile** |
| Firmicutes | Clostridiales – unknown family, unknown genus 3 | -0.59 |  |  |  |  | -0.73 |  |  | Butanoic acid |
| Firmicutes | Clostridiales; Mogibacteriaceae – unknown genus 2 | -0.33 |  |  |  |  | -0.53 |  |  | Acetic acid |
| Firmicutes | Clostridiales – Tissierellaceae – unknown genus 2 | -0.25 |  |  |  |  | -0.26 | 0.76 |  | Benzaldehide |
| Proteobacteria | Pasteurellales – Pasteurellaceae – *Haemophilus* | 0.60 |  |  |  |  | 0.68 | -0.40 |  | Octanal |
| Firmicutes | Clostridiales – Mogibacteriaceae – unknown genus 1 | 0.65 |  |  |  |  | 0.75 |  |  | Pentanal |
| Firmicutes | Clostridiales – unknown family – unknown genus 1 | 0.76 |  |  |  |  | 0.88 |  |  | Hexanal |
| Firmicutes | Erysipelotrichales – Erysipelotrichaceae – unknown genus 3 |  | -0.65 |  |  |  |  | -0.70 |  | Heptanal |
| Firmicutes | Clostridiales – Tissierellaceae – *Peptoniphilus* |  | -0.43 |  |  |  |  | -0.60 |  | Nonanal |
| Firmicutes | Clostridiales – Peptostreptococcaceae – unknown genus 3 |  | -0.34 |  |  |  |  | 0.76 |  | Dtrisulfide |
| Firmicutes | Clostridiales – Tissierellaceae – GW-34 strain 3 |  | 0.47 |  |  |  |  | 0.77 |  | Ddisulfide |
| Actinobacteria | Solirubrobacterales – Conexibacteraceae – unknown genus |  | 0.58 |  |  |  |  |  |  |  |
| Firmicutes | Clostridiales – Tissierellaceae – *Parvimonas* |  | 0.65 |  |  |  |  |  |  |  |
|  | **Explained Variance (%)** | **6.45** | **5.96** |  |  |  | **20.97** | **16.89** |  | **Explained Variance (%)** |
|  |  |  |  |  |  |  |  |  |  |  |
| PARASITISM OF FEMALES | | | | | | | | | | |
|  |  | **FEMALE** | |  |  |  |  |  |  |  |
|  |  | **SECRETIONS** | |  |  |  |  |  |  |  |
|  |  | **PC4** |  |  |  |  |  |  |  |  |
| **Phylum** | **Bacterial Orden – Family – Genus** | **BSF** |  |  |  |  |  |  |  |  |
| Firmicutes | Clostridiales – Clostridiaceae – Clostridium strain 2 | -0.71 |  |  |  |  |  |  |  |  |
| Firmicutes | Clostridiales – Clostridiaceae – Clostridium strain3 | -0.51 |  |  |  |  |  |  |  |  |
| Firmicutes | Clostridiales – Tissierellaceae – unknown genus 6 | -0.47 |  |  |  |  |  |  |  |  |
| Firmicutes | Clostridiales – Veillonellaceae – Dialister strain 1 | 0.41 |  |  |  |  |  |  |  |  |
| Firmicutes | Clostridiales – Tissierellaceae – unknown genus 1 | 0.59 |  |  |  |  |  |  |  |  |
| Firmicutes | Clostridiales – Clostridiaceae – Clostridium strain 4 | 0.63 |  |  |  |  |  |  |  |  |
|  | **Explained Variance (%)** | **5.98** |  |  |  |  |  |  |  |  |
|  |  |  |  |  |  |  |  |  |  |  |
| FLEDGING SUCCESS AND VARIABLES FROM THE EARLY NESTLING PERIOD | | | | | | | | | | |
|  |  |  |  |  |  |  | **NESTLINGS** | | |  |
|  |  |  |  |  |  |  | **SECRETIONS** | | |  |
|  |  |  |  |  |  |  | **PC1** | **PC2** | **PC3** |  |
|  |  |  |  |  |  |  | **VSN1** | **VSN1** | **VSN1** | **Volatile profile** |
|  |  |  |  |  |  |  | -0.92 |  |  | Hexanal |
|  |  |  |  |  |  |  | -0.89 |  |  | Heptanal |
|  |  |  |  |  |  |  | -0.88 |  |  | Pentanal |
|  |  |  |  |  |  |  | 0.71 |  |  | Acetic acid |
|  |  |  |  |  |  |  | 0.78 |  |  | Isobutiric acid |
|  |  |  |  |  |  |  | 0.84 |  |  | Butanoic acid |
|  |  |  |  |  |  |  |  | -0.90 |  | Butanoic acid ethyl ester |
|  |  |  |  |  |  |  |  | -0.84 |  | 3 methyl butanoic acid ethyl ester |
|  |  |  |  |  |  |  |  | -0.82 |  | Hexanoic acid ethyl ester |
|  |  |  |  |  |  |  |  | 0.59 | 0.49 | Nonanoic acid methyl ester |
|  |  |  |  |  |  |  |  | 0.68 |  | 4 methyl pentanoic acid methyl ester |
|  |  |  |  |  |  |  |  | 0.68 | 0.40 | Heptanoic acid methyl ester |
|  |  |  |  |  |  |  |  |  | -0.90 | Heptanoic acid |
|  |  |  |  |  |  |  |  |  | -0.88 | Hexanoic acid |
|  |  |  |  |  |  |  |  |  | -0.75 | Pentanoic acid |
|  |  |  |  |  |  |  |  |  | 0.52 | Butanoic acid methyl ester |
|  |  |  |  |  |  |  | **24.90** | **17.46** | **14.32** | **Explained Variance (%)** |
|  |  |  |  |  |  |  |  |  |  |  |
| FLEDGING SUCCESS AND VARIABLES FROM THE LATE NESTLING PERIOD | | | | | | | | | | |
|  |  | **NESTLINGS** | | | |  |  |  |  |  |
|  |  | **NEST** |  | **SECRETIONS** | |  |  |  |  |  |
|  |  | **PC3** |  | **PC1** | **PC5** |  |  |  |  |  |
| **Phylum** | **Bacterial Orden-Family-Genus** | **BM2** |  | **BSN2** | **BSN2** |  |  |  |  |  |
| Firmicutes | Lactobacillales – Lactobacillaceae – *Lactobacillus* | -0.53 |  |  |  |  |  |  |  |  |
| Firmicutes | Bacillales – Planococcaceae – *Sporosarcina* | -0.45 |  |  |  |  |  |  |  |  |
| Proteobacteria | Pseudomonadales – Pseudomonadaceae – *Pseudomonas* | -0.44 |  |  |  |  |  |  |  |  |
| Bacteroidetes | Saprospirales – Chitinophagaceae – unknown genus | 0.75 |  |  |  |  |  |  |  |  |
| Proteobacteria | Rhodobacterales – Rhodobacteraceae – *Paracoccus* | 0.76 |  |  |  |  |  |  |  |  |
| Proteobacteria | Rhizobiales – Brucellaceae – unknown genus | 0.82 |  |  |  |  |  |  |  |  |
| Firmicutes | Clostridiales – Clostridiaceae – *Clostridium* strain 1 |  |  | -0.84 |  |  |  |  |  |  |
| Firmicutes | Clostridiales – Mogibacteriaceae – unknown genus 2 |  |  | -0.57 |  |  |  |  |  |  |
| Firmicutes | Clostridiales – Peptostreptococcaceae – unknown genus |  |  | -0.35 |  |  |  |  |  |  |
| Firmicutes | Clostridiales – Clostridiaceae – *Clostridium* strain 2 |  |  | 0.46 |  |  |  |  |  |  |
| Firmicutes | Clostridiales – Mogibacteriaceae – unknown genus 3 |  |  | 0.63 |  |  |  |  |  |  |
| Firmicutes | Clostridiales – Clostridiaceae – *Clostridium* strain 3 |  |  | 0.76 |  |  |  |  |  |  |
| Firmicutes | Clostridiales – Tissierellaceae – *Helcococcus* strain 2 |  |  |  | -0.74 |  |  |  |  |  |
| Firmicutes | Clostridiales – Mogibacteriaceae – unknown genus 4 |  |  |  | -0.38 |  |  |  |  |  |
| Firmicutes | Clostridiales – Tissierellaceae – unknown genus 1 |  |  |  | -0.36 |  |  |  |  |  |
| Firmicutes | Clostridiales – Tissierellaceae – GW-34 strain 2 |  |  |  | 0.47 |  |  |  |  |  |
| Firmicutes | Erysipelotrichales – Erysipelotrichaceae – unknown genus 4 |  |  |  | 0.48 |  |  |  |  |  |
| Firmicutes | Clostridiales – Tissierellaceae – *Helcococcus* strain 1 |  |  |  | 0.75 |  |  |  |  |  |
|  | **Explained Variance (%)** | **15.00** |  | **6.97** | **5.86** |  |  |  |  |  |
